# Characterization of Non-Specific Cytotoxic Cell Receptor Protein 1: A New Member of the Lectin-Type Subfamily of F-Box Proteins

**DOI:** 10.1371/journal.pone.0027152

**Published:** 2011-11-07

**Authors:** Heini Kallio, Martti Tolvanen, Janne Jänis, Pei-wen Pan, Eeva Laurila, Anne Kallioniemi, Sami Kilpinen, Vilppu J. Tuominen, Jorma Isola, Jarkko Valjakka, Silvia Pastorekova, Jaromir Pastorek, Seppo Parkkila

**Affiliations:** 1 Institute of Biomedical Technology, University of Tampere, Tampere, Finland; 2 School of Medicine, University of Tampere, Tampere, Finland; 3 Center for Laboratory Medicine, Tampere University Hospital, Tampere, Finland; 4 Department of Chemistry, University of Eastern Finland, Joensuu, Finland; 5 MediSapiens Ltd, Helsinki, Finland; 6 Center of Molecular Medicine, Institute of Virology, Slovak Academy of Sciences, Bratislava, Slovak Republic; University of South Florida College of Medicine, United States of America

## Abstract

Our previous microarray study showed that the non-specific cytotoxic cell receptor protein 1 (*Nccrp1*) transcript is significantly upregulated in the gastric mucosa of carbonic anhydrase IX (CA IX)-deficient (*Car9^−/−^*) mice. In this paper, we aimed to characterize human NCCRP1 and to elucidate its relationship to CA IX. Recombinant NCCRP1 protein was expressed in *Escherichia coli*, and a novel polyclonal antiserum was raised against the purified full-length protein. Immunocytochemistry showed that NCCRP1 is expressed intracellularly, even though it has previously been described as a transmembrane protein. Using bioinformatic analyses, we identified orthologs of *NCCRP1* in 35 vertebrate genomes, and up to five paralogs per genome. These paralogs are FBXO genes whose protein products are components of the E3 ubiquitin ligase complexes. NCCRP1 proteins have no signal peptides or transmembrane domains. NCCRP1 has mainly been studied in fish and was thought to be responsible for the cytolytic function of nonspecific cytotoxic cells (NCCs). Our analyses showed that in humans, *NCCRP1* mRNA is expressed in tissues containing squamous epithelium, whereas it shows a more ubiquitous tissue expression pattern in mice. Neither human nor mouse *NCCRP1* expression is specific to immune tissues. Silencing *CA9* using siRNAs did not affect *NCCRP1* levels, indicating that its expression is not directly regulated by *CA9*. Interestingly, silencing *NCCRP1* caused a statistically significant decrease in the growth of HeLa cells. These studies provide ample evidence that the current name, “non-specific cytotoxic cell receptor protein 1,” is not appropriate. We therefore propose that the gene name be changed to FBXO50.

## Introduction

Carbonic anhydrase IX (CA IX) belongs to a family of mammalian α-carbonic anhydrases (CAs), zinc-containing metalloenzymes that catalyze the reversible hydration of carbon dioxide in the reaction CO_2_+H_2_O↔H^+^+HCO_3_^−^. CA IX is a dimeric transmembrane protein that has many unique features that distinguish it from other CAs [1], [2]. First, the expression of CA IX is limited to a few cell types in normal tissues, but it is highly expressed in several tumors, especially under hypoxic conditions. Furthermore, CA IX is involved in the regulation of tumor pH which makes it an attractive antitumor drug target [3]. Second, CA IX is the only member of the CA family that has a proteoglycan (PG) domain, which may contribute to cell adhesion, in addition to a catalytic CA domain [4]. Third, CA IX has a role in cell proliferation; this was demonstrated by the generation of CA IX knockout mice [5]. These mice showed gastric hyperplasia of the glandular epithelium with numerous cysts. In a recent study, we investigated the effect of CA IX deficiency on whole-genome gene expression in gastric mucosa using microarrays [6]. Several candidate genes were identified whose altered expression could explain the abnormal cell lineage phenotype in *Car9^−/−^* gastric mucosa. In subsequent studies, we focused on three poorly characterized genes that have only been the subject of a few published reports. One of these genes, non-specific cytotoxic cell receptor protein 1 (*Nccrp1*), was significantly overexpressed in *Car9^−/−^* gastric mucosa compared with wild-type mice.

NCCRP1 has been predicted to be a type II [7] or type III [8] membrane protein and has been cloned from catfish, zebrafish, tilapia, gilthead seabream and carp [7]–[12]. NCCRP1 is believed to be a receptor expressed in nonspecific cytotoxic cells (NCCs) that is responsible for their cytolytic function and has been suggested to consist of three domains [13].

An interesting feature of the fish NCCRP1 proteins is their homology with the F-box-only proteins. The zebrafish and catfish NCCRP1 proteins share approximately 30% identity with this subset of the F-box superfamily of proteins [12]. The homology is considered to be restricted to an F-box-associated (FBA) domain located in the C terminus, as fish NCCRP1 proteins are thought to lack the F-box motif in their N-termini. However, our analyses of its sequence and structure indicate the presence of a shorter version of the F-box domain in NCCRP1 proteins in mammals and possibly even in fish. The function of the F-box is to mediate protein-protein interactions, and F-box proteins are known to bind protein substrates for ubiquitin-mediated proteolysis [14]. F-box proteins recognize various substrates, but only members of the FBXO subfamily have the ability to bind glycoproteins [15].

The tissue distribution of *Nccrp1* mRNA has been studied in several fish species, and its expression was found to be ubiquitous. In tilapia, *Nccrp1* is expressed in all immune and non-immune tissues [9]. The relative expression level was highest in the liver, followed by the head, kidney, spleen and intestine. Lower levels were detected in the brain, gill and heart. *Nccrp1* expression was lowest in the skin. Similarly, constitutive expression of *Nccrp1* transcripts was detected in gilthead seabream [10] and carp [11]. *Nccrp1* mRNA expression has also been studied in the tetrapod animal axolotl [16]. *Nccrp1* transcripts were detected in many tissues, including the limb blastema, normal limb, skin, lung and spleen, but not in the brain or liver. High mRNA levels were detected in the spleen and regeneration blastema and in isolated blood cells.

To the best of our knowledge, this is the first study to characterize human NCCRP1. We expressed this gene in *E. coli* and analyzed its structure using biochemical methods. We also examined the expression of *NCCRP1* mRNA in human and mouse tissues. Furthermore, we have investigated the association between NCCRP1 and CA IX using siRNA technology. Our experimental and bioinformatics results contradict the previous prediction that the NCCRP1 protein is localized to the plasma membrane and point unambiguously to its function as a protein-binding F-box component of E3 ubiquitin ligases in the cytoplasm.

## Results

### Comparison of NCCRP1 and FBXO sequences

An ortholog of *NCCRP1* was identified in 35 vertebrate genomes from Ensembl ortholog tables. In fugu (*Takifugu rubripes*), two orthologs were found. We found 21 protein sequences from 20 species to be at least 90% complete. Table 1 shows the Ensembl gene identifiers for all identified orthologs, with the 20 species used in further bioinformatic analyses in bold.

**Table 1:** NCCRP1 orthologs found in Ensembl genomes.*

Species	Ensembl Gene ID
Anole Lizard (Anolis carolinensis)	ENSACAG00000011950
Armadillo (Dasypus novemcinctus)	ENSDNOG00000018735
**Bushbaby (Otolemur garnettii)**	ENSOGAG00000012185
Cat (Felis catus)	ENSFCAG00000007425
**Chimpanzee (Pan troglodytes)**	ENSPTRG00000010953
Cow (Bos taurus)	ENSBTAG00000014296
Dog (Canis familiaris)	ENSCAFG00000005593
Dolphin (Tursiops truncatus)	ENSTTRG00000014969
Elephant (Loxodonta africana)	ENSLAFG00000026532
**Fugu (Takifugu rubripes) (1 of 2)**	ENSTRUG00000006309
**Fugu (Takifugu rubripes) (2 of 2)**	ENSTRUG00000013740
**Gibbon (Nomascus leucogenys)**	ENSNLEG00000014516
**Gorilla (Gorilla gorilla)**	ENSGGOG00000028030
Guinea Pig (Cavia porcellus)	ENSCPOG00000009451
Hedgehog (Erinaceus europaeus)	ENSEEUG00000015408
Horse (Equus caballus)	ENSECAG00000018954
**Human (Homo sapiens)**	ENSG00000188505
Hyrax (Procavia capensis)	ENSPCAG00000004015
Kangaroo rat (Dipodomys ordii)	ENSDORG00000001051
**Macaque (Macaca mulatta)**	ENSMMUG00000013906
**Marmoset (Callithrix jacchus)**	ENSCJAG00000014055
**Medaka (Oryzias latipes)**	ENSORLG00000000459
**Microbat (Myotis lucifugus)**	ENSMLUG00000002162
**Mouse (Mus musculus)**	ENSMUSG00000047586
**Opossum (Monodelphis domestica)**	ENSMODG00000013456
**Orangutan (Pongo abelii)**	ENSPPYG00000009956
Panda (Ailuropoda melanoleuca)	ENSAMEG00000008646
Pika (Ochotona princeps)	ENSOPRG00000006693
Platypus (Ornithorhynchus anatinus)	ENSOANG00000015297
**Rabbit (Oryctolagus cuniculus)**	ENSOCUG00000011658
**Rat (Rattus norvegicus)**	ENSRNOG00000026244
**Stickleback (Gasterosteus aculeatus)**	ENSGACG00000020167
**Tetraodon (Tetraodon nigroviridis)**	ENSTNIG00000006031
**Wallaby (Macropus eugenii)**	ENSMEUG00000001939
**Xenopus tropicalis**	ENSXETG00000005319
**Zebrafish (Danio rerio)**	ENSDARG00000035326

*Twenty-one genes with at least 90% complete predicted protein products are shown in boldface.

**Figure 1:**
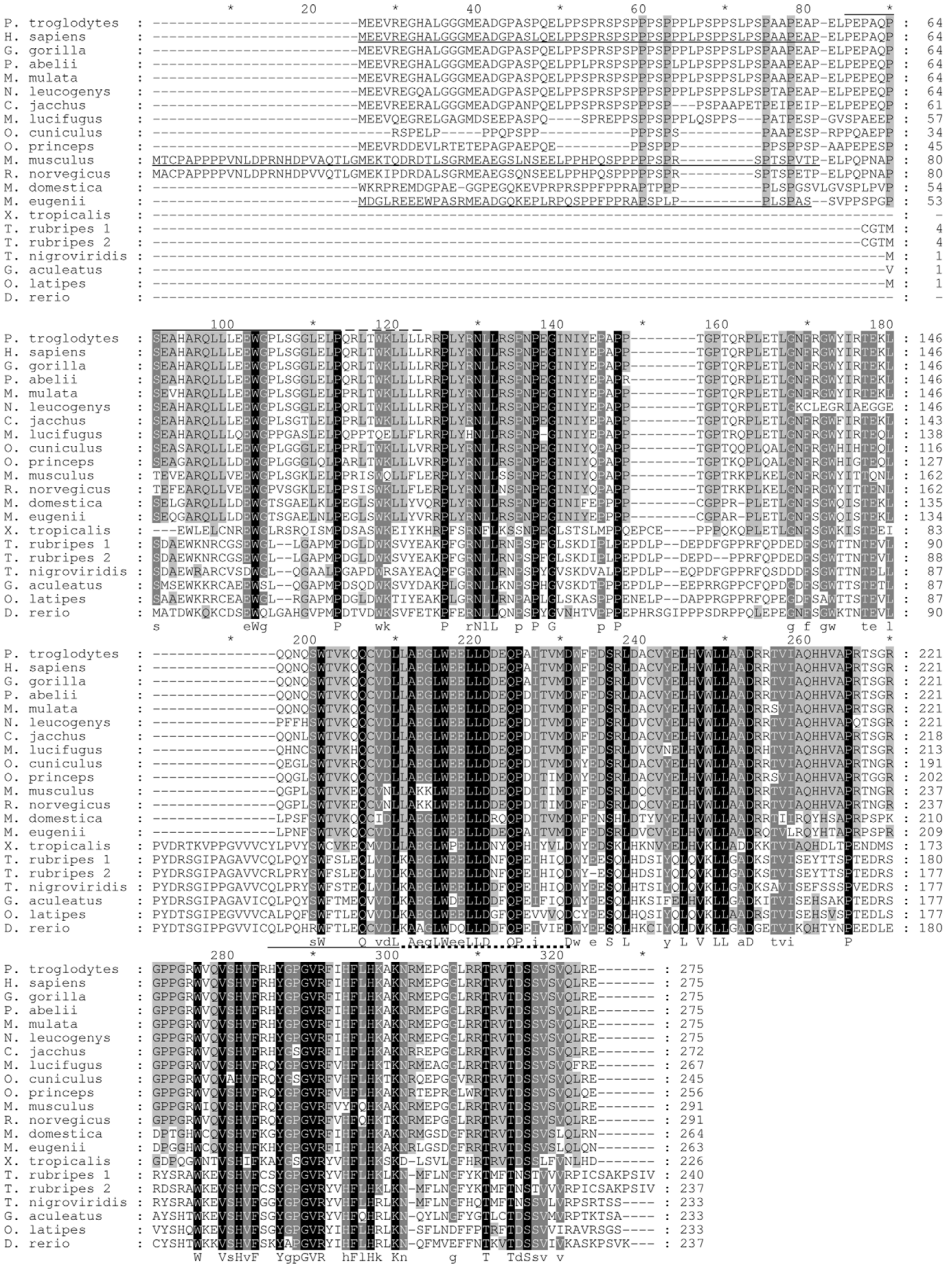
Alignment of 21 NCCRP1 protein sequences from 20 species. Shading indicates conservation, with more strongly conserved positions shaded in darker colors. Uppercase letters under the alignment give the identity of fully conserved residues, and lowercase letters show the consensus residue of partially conserved positions. Underlined sequences in the N-terminal region are predicted to be disordered in the three species studied. The line over the sequence (columns 85 to 123) indicates the predicted F-box domain. The solid line (cols. 85 to 113) indicates an alternative model (see “Analysis of protein domains”). Underlining of the bottom consensus line indicates the location of the peptides discussed in “Modeling of suggested active peptides” and studied in previous work [8]. Solid line: 16-mer peptide; Solid line plus dotted line: 37-mer peptide.

Multiple sequence alignment of 21 NCCRP1 proteins in Fig. 1 shows that the mouse and rat sequences in Ensembl are either 25 residues longer at the N-terminus, or the real start codon is the one corresponding to M26. Mammalian sequences are characterized by a proline-rich N-terminal domain of approximately 60 residues, whereas fish sequences show shorter insertions around columns 150 and 190 in the alignment. NCCRP1 from the frog *X. tropicalis* has short insertions similar to the fish sequences, but the N-terminus is incomplete, so the presence of the N-terminal domain remains inconclusive.

A major portion of the N-terminal domain in mammals is predicted to be disordered. Prediction analysis of the human, mouse and wallaby sequences yielded disordered domains of 70, 57 and 49 residues, respectively. These regions are underlined in Fig. 1, which shows the end point of the disordered regions to be very close to the end of the additional sequence seen in mammals. There is an analogous Glu- and Ala-rich N-terminal domain in the PEST domain of FBXO2 that also seems to be disordered. The N-terminal 47 residues show no electron density in the crystal structures of mouse FBXO2 (2E31, 2E32, 2E33) [17], and the disorder prediction is consistent with the 41 N-terminal residues predicted to be disordered.

The human paralogs of *NCCRP1*, as shown by the Ensembl comparative genomics tools and Blast searches, are the genes *FBXO2*, *FBXO6*, *FBXO17*, *FBXO27* and *FBXO44*. The protein products of these five genes are components of E3 ubiquitin ligase complexes, and they define a lectin subfamily of ubiquitin ligases [15]. In humans, the genes *FBXO2*, *FBXO6* and *FBXO44* are contiguous genes on chromosome 1 (at 11.70 to 11.74 Mb), and *FBXO17*, *FBXO27* and *NCCRP1* are on chromosome 19 (at 39.4 to 39.7 Mb). The protein sequence identity of NCCRP1 with the other five lectin-type FBXO proteins ranges from 31% to 36% in the last 180 residues, and the Blast E values range from 10^−27^ to 10^−18^, clearly indicating that these proteins share homology and common ancestry.

In the available fish genomes, we only found orthologs to *FBXO2*, *FBXO44* and *NCCRP1*. Interestingly, the zebrafish genome carries a cluster of ten copies of *FBXO44* orthologs on chromosome 23.

### Analysis of protein domains

By Interpro scan, all 21 protein sequences had domain matches to “Fbox-associated” (FBA, IPR007397) and “galactose-binding domain-like” (IPR008979) patterns in the ∼200 C-terminal residues in fish and frog species and ∼180 residues in mammalian species. Only the protein products of *NCCRP1* and the above five *FBXO* gene loci are annotated to contain an FBA domain (InterPro entry IPR007397), and no other human proteins were found in Blast searches when NCCRP1 or the FBA domain of FBXO2 were used as queries. The products of these five human *FBXO* genes also contain a cyclin-like F-box domain pattern (InterPro entry IPR001810) in the N-terminal region that spans 50 residues or less, but none of the NCCRP1 proteins match this pattern.

**Figure 2:**
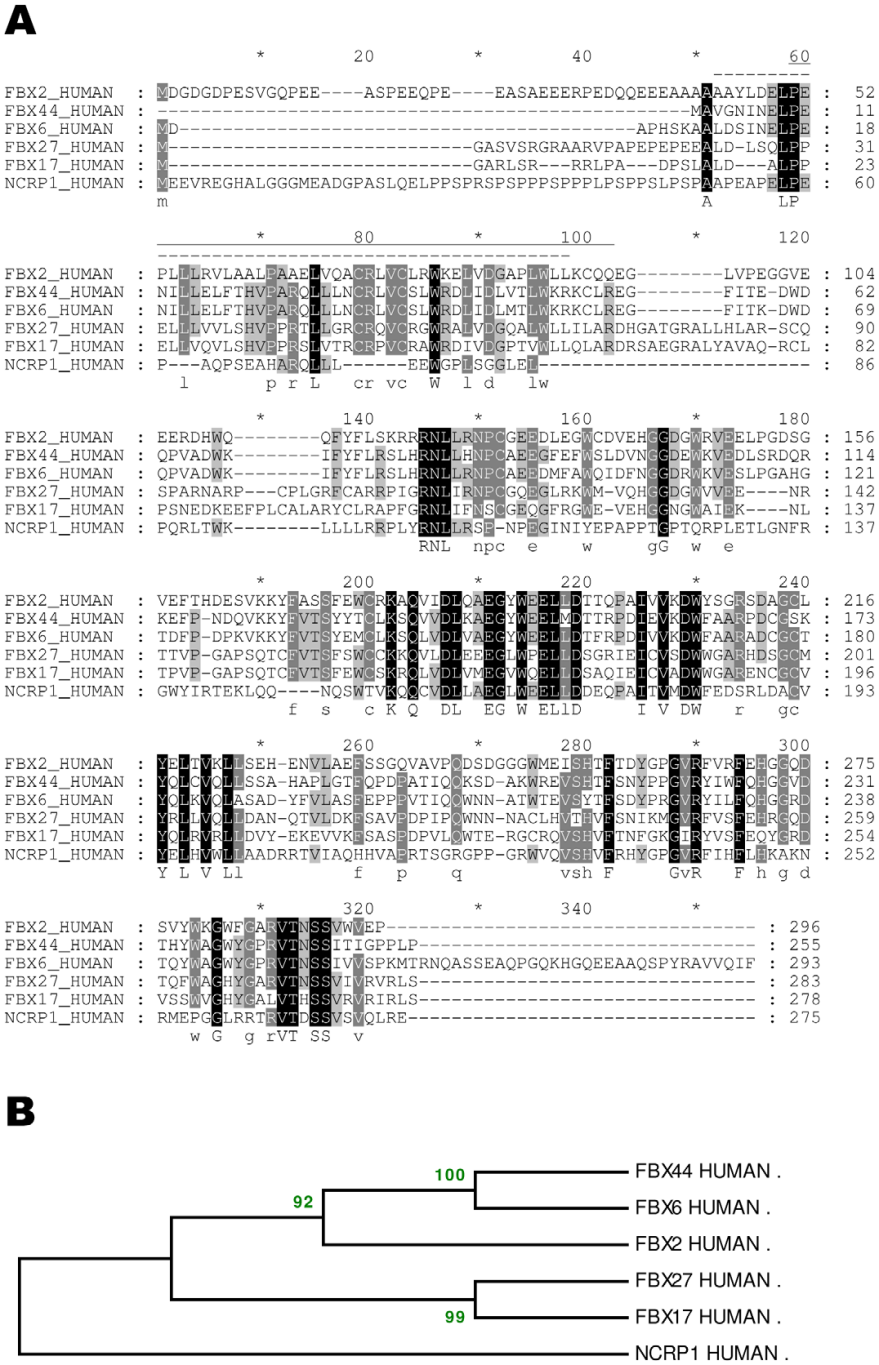
Alignment of human NCCRP1 with its closest five paralogs and their phylogenetic tree. (A) Shading and consensus lettering were used as described in Fig. 1. Dotted line over the sequence: F-box domain, cyclin-like, as defined in human FBXO2 by InterPro. Solid line over the sequence: F-box domain as defined by compact alpha-helical folding in PDB entry 2E31 [17]. Line under the sequence: the sugar-binding domain as defined previously [17]. (B) Phylogenetic tree with bootstrap support percentages for each node.

Fig. 2 shows the alignment of human NCCRP1 with these five FBXO proteins and their phylogenetic tree. Although there was no match to the cyclin-like F-box domain pattern in the NCCRP1 sequence, 12 residues are conserved between human NCCRP1 and at least three other FBXO proteins within the F-box domain region (lines on top of the alignment in Fig. 2). The main differences in this region are found in three indels, in which NCCRP1 has shorter sequences and lacks many conserved residues, most notably the signature pattern CRxVC. To test whether these deletions would be compatible with the F-box domain structure, we visualized them in the known 3D structure of mouse FBXO2 [17].

**Figure 3:**
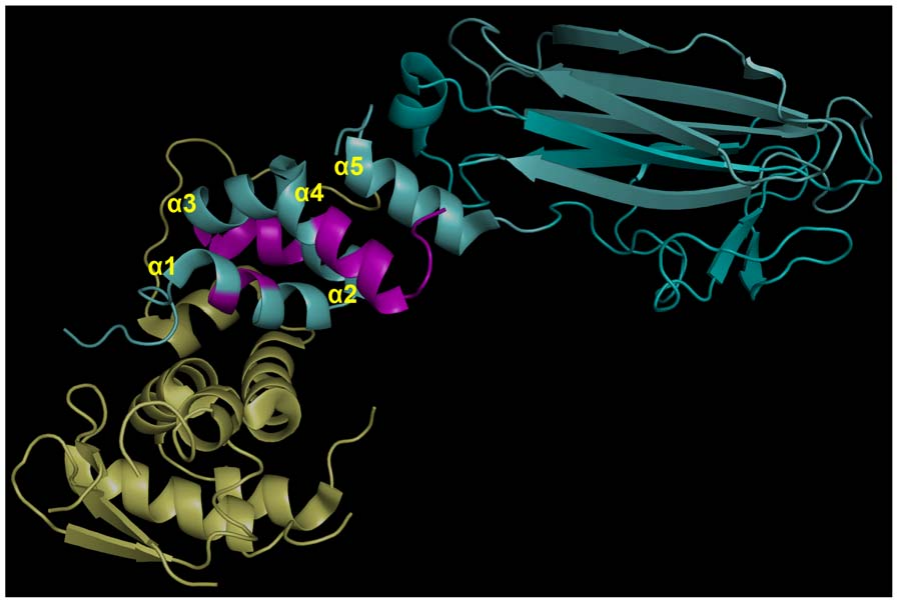
Structure of mouse FBXO2 (cyan) complexed with SKP1 (yellow). PDB entry 2E31 [17]. The regions of FBXO2 shown in purple correspond to parts that would be deleted from a model of the F-box domain of human NCCRP1. The first 46 residues and residues 104–108 of FBXO2 are not resolved in the structure. The sugar-binding site (SBD) is at the extreme right in this image. Alpha helices of FBXO2 are labeled at the N-end of each helix, as described previously [17].

Fig. 3 shows the sequences to be deleted in purple. They constitute one complete helical turn in alpha helix α1 (helices named as described previously [17]), one helix turn in α2 and part of the α2′ loop, the end of helix α4, and the major part of the linker before α5, including the flexible residues 104 to 108 that are missing from the structure between the open ends (purple and cyan). These deletions could be modeled in a shorter version of the F-box fold while retaining the positions of α3 and α4 and the sugar-binding domain (SBD), as in the structure 2E31 in PDB, by linking α5 to the truncated α4 and shifting the right-hand ends of helices α1 and α2 to the left. Alternatively, the SBD could be moved to bring α5 into the position of α4, which would still maintain the relative orientation between the F-box and the SBD. Fig. 1 shows the extent of the F-box domain in these two models. The solid line over the alignment indicates the version in which α4 is truncated, whereas the dotted line indicates the fusion of α5 into the F-box domain. In conclusion, we believe that the region preceding the SBD in human and other mammalian NCCRP1 proteins is indeed folded as an F-box domain.

In fish NCCRP1 sequences, the F-box domain is not detected (by matching to the F-box pattern in InterPro), which is not surprising because the fish sequences start in the middle of the F-box region (Fig. 1). However, because the fish sequences only lack six residues or less from the beginning of the structurally defined F-box domain (see line at the top of the alignment in Fig. 1), a version with a shortened initial helix α1 might exist. This remains speculative because the fish and mammalian sequences are not highly conserved in the putative F-box region.

TMHMM predicted that there are no transmembrane helices in any of the studied 21 NCCRP1 ortholog proteins. SignalP predicted no signal peptides in any of the proteins, and TargetP predicted a non-mitochondrial, non-secreted localization for all proteins. The predictions for the mouse and rat sequences were run with alternative start sites: residue 1, as in Ensembl proteins, and residue 26, consistent with the translation start site of other mammalian sequences. All of these results indicate the absence of transmembrane domains and cytoplasmic localization of NCCRP1 in all of the studied species.

Jaso-Friedmann et al. [12] reported Box-1 motifs in zebrafish and catfish NCCRP1 proteins. We did not detect these motifs in the NCCRP1 proteins we analyzed, and we disagree with their finding because they took the liberty of reversing the sequences, which does not make sense for peptides.

### Modelling of suggested active peptides

**Figure 4:**
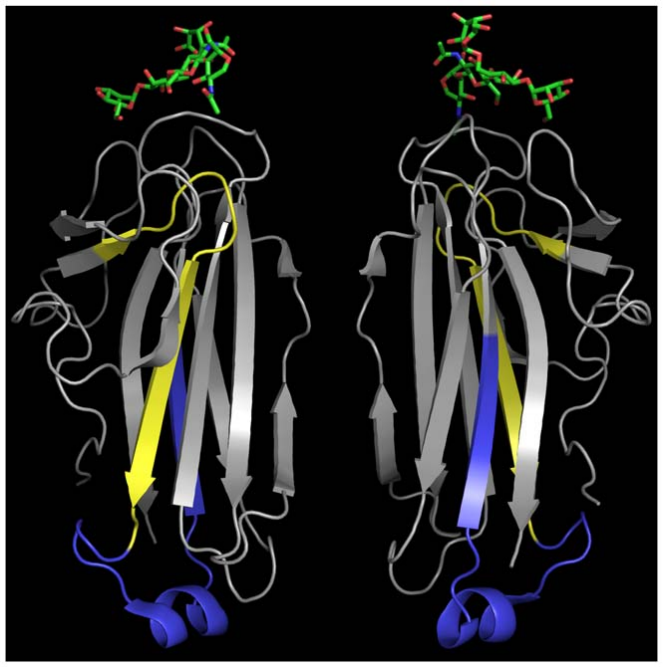
Structure of the sugar-binding domain of mouse FBXO2 complexed with an oligosaccharide of bovine ribonuclease A. PDB entry 2E33 [17]. Two views rotated 180 degrees vertically are shown. The protein part of ribonuclease A is omitted. The coloring indicates the positions homologous to the NCCRP1 peptides discussed in “Modeling of suggested active peptides” and studied in previous work [8], [13]. Yellow: 16-mer peptide; yellow plus blue: 37-mer peptide.

Because the NCCRP1 proteins could not be located on the cell surface, as would be expected for the proteins to function as antigen receptors [7], we next asked whether there were any structural explanations for the previously reported [8], [12] cytotoxicity-inhibiting activity of NCCRP1-derived peptides (a more potent 16-mer and a less potent 37-mer). The location of these two overlapping peptides is shown under the alignment in Fig. 1. Fig. 4 shows the location of the 16-mer in yellow and the 37-mer in yellow plus blue, extrapolated into the structure of mouse FBXO2 based on the sequence alignment. These peptides contain long beta-strand elements coming from the amphiphilic beta sheets, which have one side of mainly hydrophobic residues (facing the protein core). Randomized peptides with same residues would be highly unlikely to have such an organized amphiphilic surface.

### Production, purification and characterization of recombinant human NCCRP1

**Figure 5:**
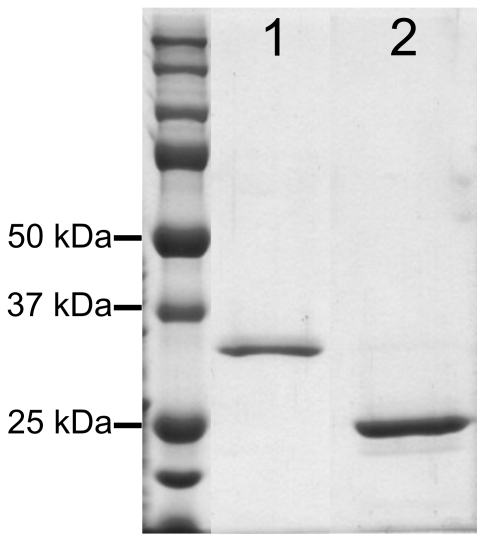
SDS-PAGE of the human recombinant NCCRP1 protein produced in *E. coli*. Lane 1 shows the 30-kDa NCCRP1 eluted from the affinity chromatography column after thrombin treatment. In lane 2, 26-kDa GST is shown.

The human *NCCRP1* cDNA was cloned into the expression vector pGEX-4T-1 and expressed as a fusion protein with GST. The recombinant NCCRP1 protein was characterized using SDS-PAGE based on its apparent size. After purification by affinity chromatography and digestion with thrombin, NCCRP1 appeared as polypeptide of approximately 30 kDa after SDS-PAGE (Fig. 5, lane 1). Fig. 5 also shows the 26-kDa GST band (lane 2).

**Figure 6:**
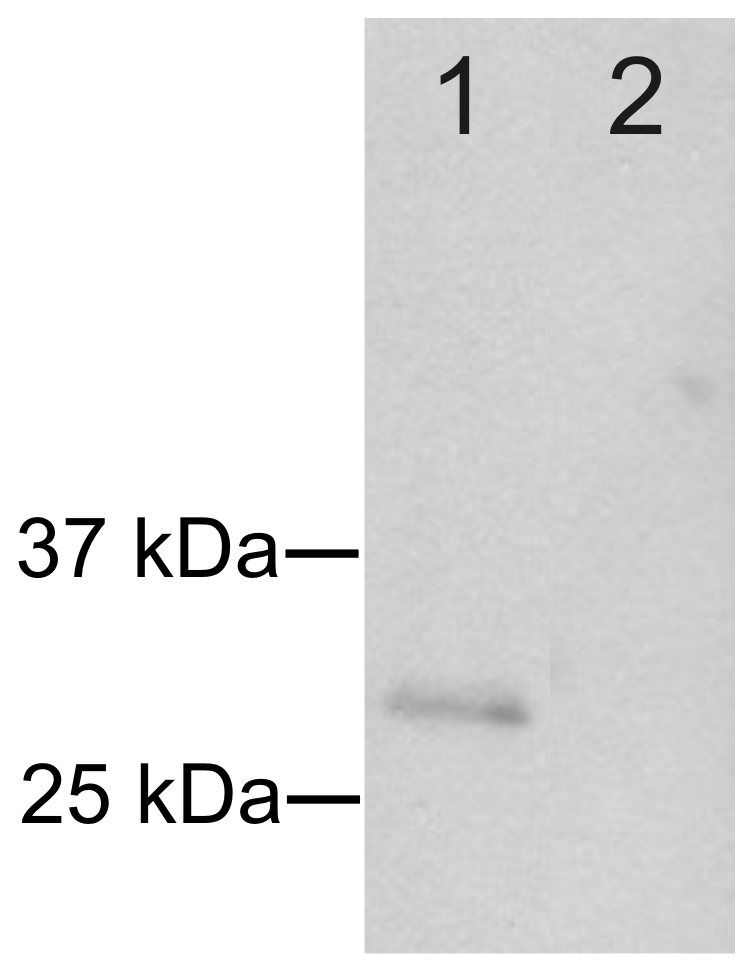
Western blot analysis of the antibody produced against NCCRP1. Lane 1 shows a single band confirming the expected reactivity of the antibody towards NCCRP1 protein. The control experiment using normal rabbit serum is negative (lane 2).

Western blotting was used to confirm the reactivity of the antibody produced against NCCRP1. Fig. 6 shows a single band for NCCRP1 in lane 1, whereas the control blot using normal rabbit serum was negative (Fig. 6, lane 2).

### Mass spectrometry

**Figure 7:**
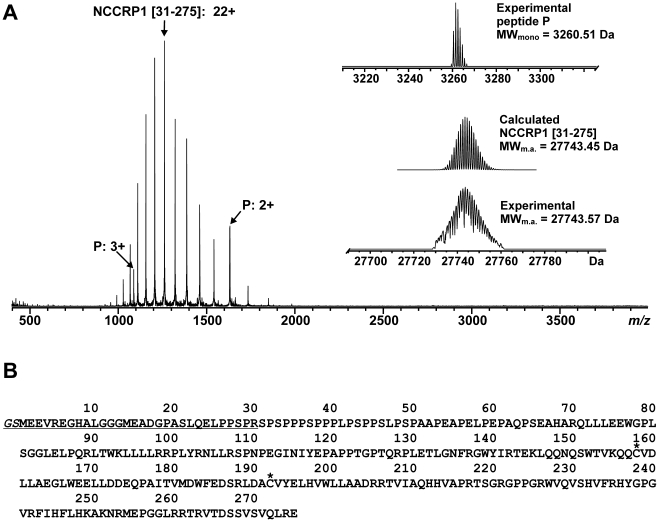
Mass spectrometry analysis of NCCRP1. (A) ESI FT-ICR mass spectrum of NCCRP1 sample measured in an acetonitrile/water/acetic acid (49.5∶49.5∶1.0, v/v) mixture. The sample contained an NCCPR1 [31–275] fragment (charge state 22+ denoted) and a peptide P (charge states 2+ and 3+ denoted), corresponding to the first 30 residues with an additional Gly-Ser in the N-terminus due to the used expression vector construct. The charge-deconvoluted mass spectrum in the inset shows calculated and experimental isotopic distributions for the NCCRP1 [31–275] fragment (with the most abundant masses indicated), as well as experimental isotopic distribution for the peptide P with a monoisotopic mass of 3260.15 Da. (B) Primary sequence of NCCRP1. The residues corresponding to the peptide P are underlined and cysteines (Cys158, Cys192) are marked with asterisks.

Upon buffer exchange, the NCCRP1 protein sample slightly precipitated, and therefore, mass analyses were conducted from both the precipitate and supernatant. A small amount of the precipitate was dissolved in 100 µl of 10 mM ammonium bicarbonate buffer, pH 8.5, further diluted with acetonitrile/water/acetic acid (49.5∶49.5∶1.0, v/v) mixture and directly analyzed with ESI FT-ICR mass spectrometry. From this analysis, the precipitate was found to contain a 27-kDa protein as well as a small 3-kDa peptide (Fig. 7A). The most abundant isotopic mass of the protein (27743.57 Da) was matched against the NCCRP1 sequence and a reasonable match was found with the fragment containing residues 31–275 (theoretically 27743.45 Da), indicating the presence of one intra-molecular disulfide (Cys158-Cys192) in the protein structure (see inset in Figure 7A). The monoisotopic mass of the observed peptide P (3260.51 Da) was also matched against the NCCRP1 sequence, and it was found to correspond to the first 30 residues of NCCRP1 with an additional Gly-Ser in the N-terminus (theoretically 3260.52 Da). These amino acids are attributable to the pGEX-4T-1 expression vector construct (Fig. 7B).

To further identify the produced protein, a database search for the trypsin-digested protein was performed. An aliquot of 20 µl of the tryptic digest sample was further diluted with 150 µl of an acetonitrile/water/acetic acid (49.5∶49.5∶1.0, v/v) mixture and analyzed directly. The digestion resulted in 18 identified tryptic peptides within an average mass error of 6 ppm, covering 75% of the NCCRP1 sequence. A search against the SwissProt database gave an unambiguous hit for human NCCRP1 (NCRP1_HUMAN) with a Mowse score of 370 (Figure S1).

Mass spectra measured from the supernatant instead revealed the same 3-kDa peptide that was detected from the precipitate as well as a small amount of the NCCRP1 [31–275] fragment. In addition, small amounts of some larger, currently unidentified 7–10 kDa polypeptides were detected (data not shown).

### Localization of NCCRP1 in HeLa cells

**Figure 8:**
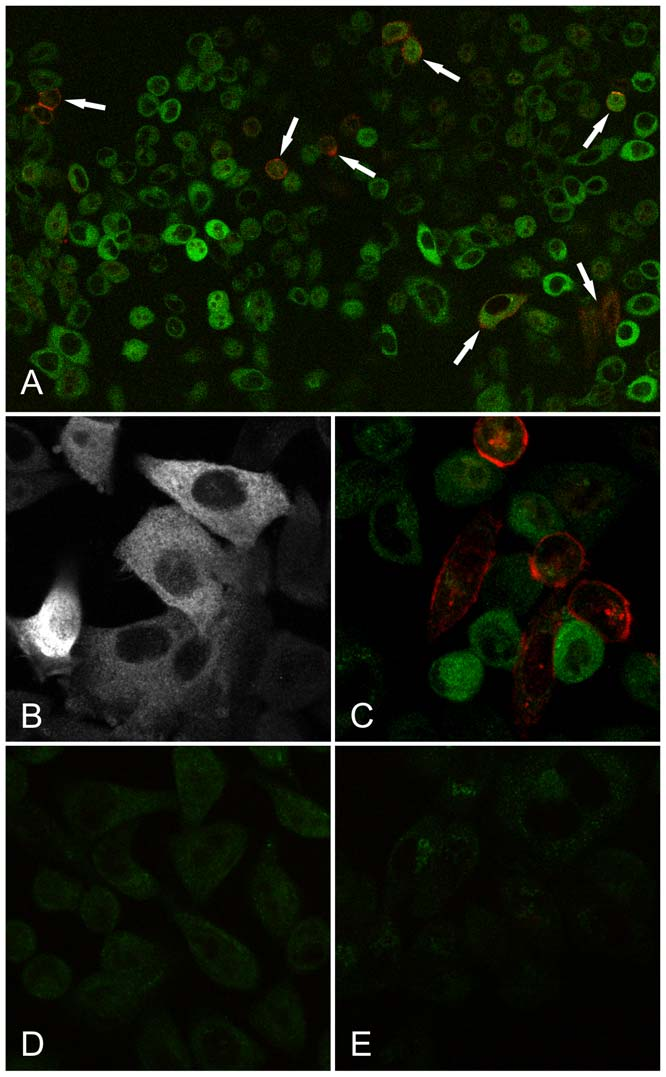
Subcellular localization of NCCRP1 in HeLa cells by immunocytochemistry. (A, B) Staining for NCCRP1 is detected in the cytosol as shown in green. (A, C) CA IX is expressed on the cell membrane as shown in red. (A; arrows) Only a few HeLa cells expressed both proteins at the same time. (D) Control immunostaining using 30 µg of blocking NCCRP1 recombinant protein and anti-NCCRP1 serum together gives only a very faint staining. (E) Other control staining using pre-immune serum instead of the anti-NCCRP1 serum shows a barely detectable signal.

The subcellular localization of NCCRP1 was examined in HeLa cells by immunocytochemistry. The protein was detected in the cytoplasm, as shown in green in Fig. 8. In contrast, CA IX was localized at the cell membrane, as expected (Fig. 8A and C, red). Interestingly, these two proteins were only rarely detected in the same cell (Fig. 8A, arrows). Control immunostaining using 30 µg of blocking NCCRP1 recombinant protein and anti-NCCRP1 serum resulted in only very faint staining, confirming the specificity of the antiserum (Fig. 8D). A second control using pre-immune serum instead of the anti-NCCRP1 serum showed virtually no staining (Fig. 8E).

### *NCCRP1* expression in human tissues

**Figure 9:**
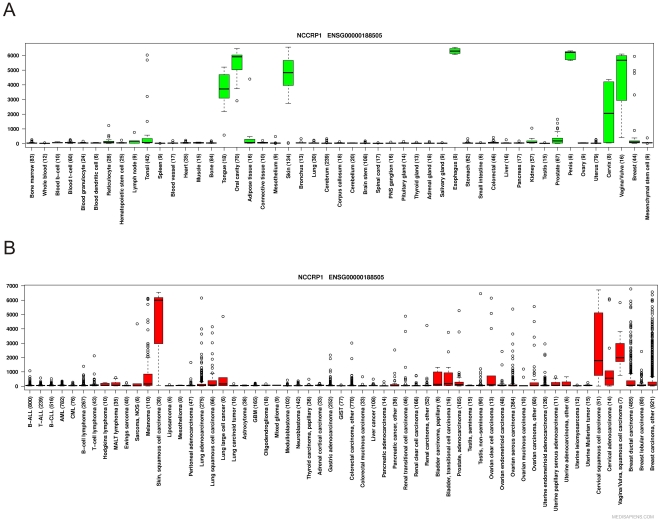
Data for human *NCCRP1* mRNA expression from the MediSapiens database. (A) Boxplot analysis of the *NCCRP1* expression levels across healthy human tissues. (B) Boxplot analysis of the *NCCRP1* expression levels across a large panel of cancer tissues. Among the normal tissues with the highest *NCCRP1* expression are the esophagus, oral cavity, skin, tongue and reproductive organs. In cancerous tissues, the expression profile of *NCCRP1* is relatively narrow, with the highest expression seen in the squamous cell carcinomas of the skin and cancers of the female reproductive organs. The number of samples in each category is shown in parenthesis. The box refers to the quartile distribution (25–75%) range, with the median shown as a black horizontal line. The whiskers extend to 1.5 times the interquartile range from the edges of the box, and any data points beyond this are considered outliers and marked by open circles.

The IST database system developed by MediSapiens Ltd was used to obtain the data for *NCCRP1* mRNA expression in human normal and cancer-derived tissues (Fig. 9A and B). The expression pattern of *NCCRP1* was relatively narrow in both normal and cancerous tissues. The normal tissues with the highest signal were the esophagus, oral cavity, skin, tongue and male and female reproductive organs. The cancer-derived tissues with high *NCCRP1* expression included the squamous cell carcinoma of the skin and cancers of the female reproductive organs.

### *Nccrp1* expression in mouse tissues

**Figure 10:**
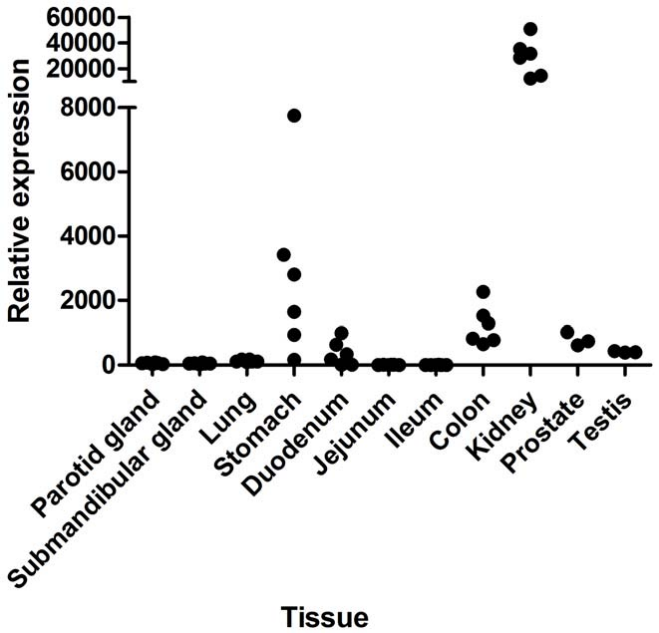
The *Nccrp1* mRNA expression in mouse tissues by QRT-PCR. *Nccrp1* is present in all the tissues studied. The expression of *Nccrp1* is highest in the kidney and moderate in the stomach, colon, duodenum and prostate. In the other tissues *Nccrp1* expression is modest. Normalized median values calculated from the technical triplicates are shown.

*Nccrp1* mRNA expression was studied in several mouse tissues by QRT-PCR. *Nccrp1* transcript was expressed in all tissues studied and the signal was highest in the kidney (Fig. 10). The expression was moderate in the stomach, colon, duodenum and prostate. In other tissues, *Nccrp1* was expressed to a lesser degree but signal was still clearly detectable.

### The effect of growth factors and deferoxamine mesylate on *NCCRP1* mRNA expression

**Figure 11:**
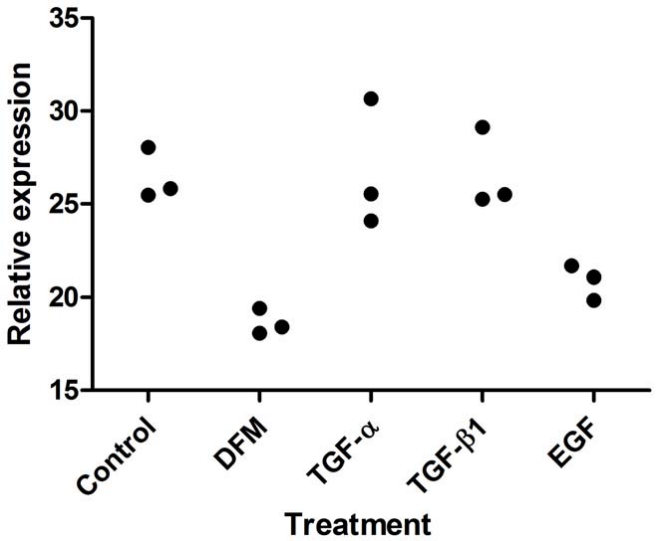
The effect of different treatments on *NCCRP1* levels in HeLa cells. The treatments included deferoxamine mesylate (DFM), transforming growth factor-alpha (TGF-α), transforming growth factor-beta 1 (TGF-β1) and epidermal growth factor (EGF). Deferoxamine mesylate and EGF treatments moderately decreased the expression of *NCCRP1* (1.4-fold and 1.2-fold, respectively). The normalized values of triplicate experiments as detected by QRT-PCR are shown.

The influence of growth factors and deferoxamine mesylate on *NCCRP1* transcript levels was studied in HeLa cells using QRT-PCR. The basal level of *NCCRP1* expression was relatively high in these cells and neither treatment had a major effect on its expression. After treatment with deferoxamine mesylate, which is commonly used to induce a hypoxia-like response, the expression of *NCCRP1* decreased moderately (1.4-fold), as shown in Fig. 11. In contrast, deferoxamine mesylate treatment induced *CA9* expression 4-fold, as expected (data not shown). Stimulation with EGF decreased *NCCRP1* expression levels but the decline was small (1.2-fold). Other treatments did not have any effect on *NCCRP1* mRNA levels.

### Expression of *NCCRP1* and *CA9* in human pancreatic and breast cancer cell lines

**Figure 12:**
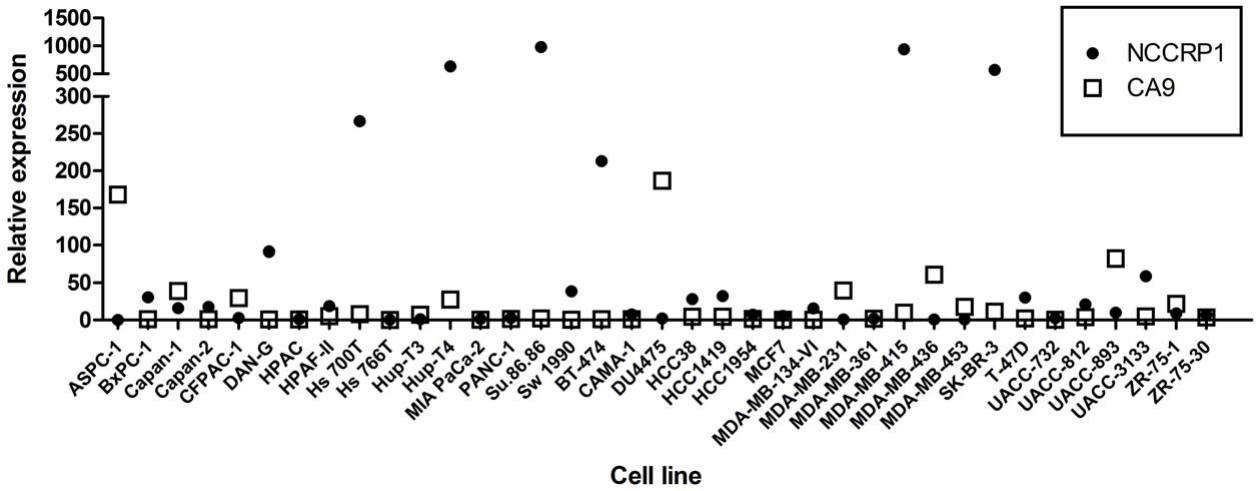
Screening of *NCCRP1* and *CA9* mRNA expression in cancer cell lines. 16 human pancreatic (AsPC-1 to SW 1990) and 21 breast (BT-474 to ZR-75-30) cancer cell lines were screened using QRT-PCR. The expression of *NCCRP1* is highest in the Su.86.86, Hup-T4 and Hs700T pancreatic cancer cell lines and the MDA-MB-415, SK-BR-3 and BT-474 breast cancer cell lines. *CA9* is most highly expressed in the DU4475 breast cancer and AsPC-1 pancreatic cancer cell lines. The expression levels of these genes typically show a reciprocal pattern: high simultaneous expression is observed in none of these cell lines. The normalized values are shown.

The expression of *NCCRP1* and *CA9* was measured by QRT-PCR in 16 pancreatic cancer cell lines and 21 breast cancer cell lines. *NCCRP1* showed the highest expression in the Su.86.86, Hup-T4 and Hs700T pancreatic cancer cell lines and the MDA-MB-415, SK-BR-3 and BT-474 breast cancer cell lines (Fig. 12). In other cell lines, *NCCRP1* was expressed at moderate or low levels. In general, the expression of *CA9* was lower than *NCCRP1* in the studied cell lines. *CA9* had the strongest signal in the DU4475 breast cancer and AsPC-1 pancreatic cancer cell lines. Its expression was moderate in a few cell lines and very low in several others. *NCCRP1* and *CA9* showed reciprocal expression patterns in some cell lines; i.e., when *NCCRP1* expression was high *CA9* expression was low or absent, and vice versa.

### *NCCRP1* and *CA9* silencing

*CA9* was silenced in the human glioblastoma cell line, U373, which has a high basal level of *CA9* expression. Efficient downregulation of mRNA expression (up to 93% reduction compared with luciferase control siRNA-treated cells) was detected for the *CA9* gene after siRNA treatment (data not shown). Silencing of *CA9* was monitored until 144 h after transfection and remained efficient at that time point. The mRNA level of *NCCRP1* was studied in U373 cells after *CA9* silencing to examine whether *NCCRP1* was directly regulated by *CA9* expression. The expression of *NCCRP1* was below the detection limit of QRT-PCR at the basal level in U373 cells and did not change after the silencing of *CA9*.

**Figure 13:**
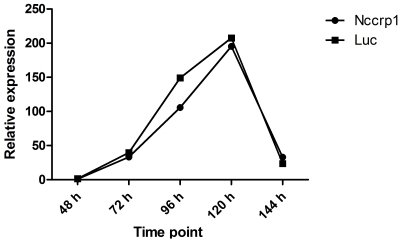
*CA9* expression after silencing of *NCCRP1* in HeLa cells. The mRNA expression of *CA9* was monitored for 144 h after the silencing of *NCCRP1*. No change was observed in *CA9* expression with *NCCRP1* siRNA compared with *luciferase* (Luc) control siRNA. The normalized median values calculated from the three replicates are shown.

*NCCRP1* silencing was performed in two cell lines, HeLa and Su.86.86, and the effect of silencing on the expression level of *CA9* was studied. The silencing of *NCCRP1* was efficient in both cell lines (up to 91% and 83% reduction compared with luciferase control siRNA-treated cells for HeLa and Su.86.86 cells, respectively). In HeLa cells, the silencing of *NCCRP1* was monitored until 144 h after transfection and remained efficient. *CA9* mRNA levels were not affected by *NCCRP1* silencing (Fig. 13). The expression of *CA9* steadily increased until 120 h after transfection, after which it decreased dramatically. In Su.86.86 cells, *CA9* expression was also unaffected by the silencing of *NCCRP1* (data not shown).

### Effect of *NCCRP1* silencing on cell growth

**Figure 14:**
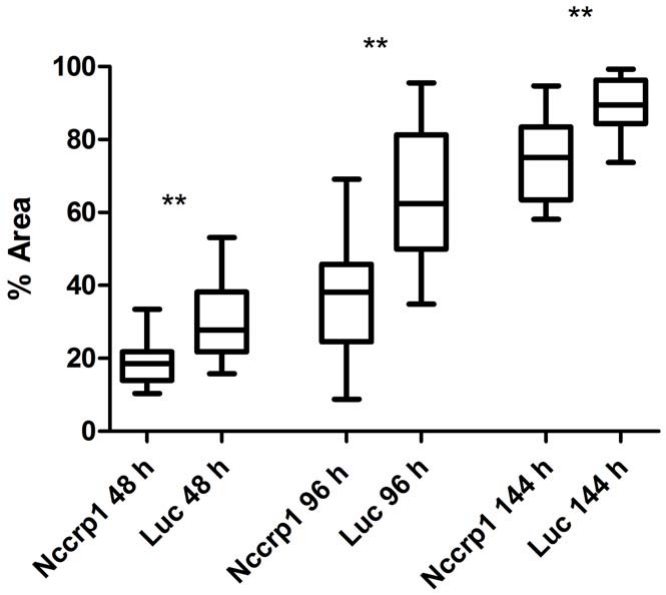
The effect of *NCCRP1* silencing on cell proliferation in HeLa cells as analyzed by ImageJ. Cell growth was studied at 48, 96 and 144 h after transfection. The silencing of *NCCRP1* caused a statistically significant decrease in cell proliferation at every time point. Statistically significant differences relative to luciferase (Luc) control siRNA were determined. ** p<0.01.

The effect of *NCCRP1* silencing on cell growth was studied in HeLa cells at 48, 96 and 144 h after transfection, and the analysis was performed using ImageJ. Silencing of *NCCRP1* led to a statistically significant decrease in HeLa cell proliferation at every time point (Fig. 14).

## Discussion

According to our previous cDNA microarray analysis, carbonic anhydrase IX-deficient mice showed significantly upregulated expression of *Nccrp1* in the stomach, which is the most abundant site of CA IX expression in normal mice. This finding led us to investigate possible connections between these two genes/proteins and to further assess the structure, regulation and function of the NCCRP1 protein. Our bioinformatic analyses clearly linked NCCRP1 protein to the FBXO gene family. The FBA domain in the FBXO2, FBXO6, FBXO17 and FBXO27 proteins is a lectin domain, and these proteins have been experimentally shown to bind various glycans [15]. The presumed role of these proteins is to bind misfolded and retrotranslocated glycoproteins in the cytoplasm for ubiquitin conjugation leading to proteasomal degradation [15]. The fact that this family of lectin-type F-box proteins (among the dozens of different F-box proteins found in ubiquitin ligase complexes) comprises the closest paralogs of NCCRP1 gives us a strong reason to predict that NCCRP1 functions as a protein-binding component in ubiquitin ligase complexes. Whether it actually binds glycans, similar to FBXO2, FBXO6, FBXO17 and FBXO27, or lacks the ability to bind sugar, like FBXO44 [15], remains to be tested experimentally. However, sequence alignments and structural interpretations (Figs. 1, 2, 3) suggest that NCCRP1 is a sixth member of the lectin-type FBXO gene family, and we therefore propose that the gene name be changed to FBXO50 (the number may change based on the decision of the Human Gene Nomenclature Committee, http://www.genenames.org/).

Jaso-Friedmann et al. [12] stated that NCCRP1 does not contain an F-box and is therefore an exception in this subfamily. However, our analysis points to the existence of an F-box in mammalian NCCRP1 proteins, and at least the remnants of one in the fish sequences, so the name FBXO50 would be appropriate.

The “F-box associated” domain occurs in the human proteome only in the six gene products discussed in this paper. This domain could also be renamed to reflect its known functions, e.g. F-box associated lectin(-like) domain or ubiquitin ligase lectin(-like) domain.

To our surprise, mass spectrometry analysis indicated the presence of a truncated protein in the NCCRP1 sample after purification and buffer exchange, which corresponded to the residues 31–275 of the full-length polypeptide. However, the peptide corresponding to the first 30 residues was also detected, which suggests that the N-terminal region had been cleaved after the protein production, probably during purification or desalting steps. Protein precipitation was also observed upon buffer-exchange but it is not known whether this is related to the observed N-terminal cleavage or change in the buffer conditions. The reason for the intrinsically weak N-terminal region of the NCCRP1 protein is unknown and will be investigated further. Based on our sequence analyses, the N-terminal proline-rich region of mammalian NCCRP1 proteins is predicted to be disordered, which might contribute to the N-terminal truncation. Mass analysis of the NCCRP1 [31–275] fragment also indicated that there is an intra-molecular disulfide between Cys158 and Cys192.

None of the NCCRP1 sequences contain any putative signal sequences or transmembrane domains, which rules out a membrane location. Accordingly, we showed using immunocytochemistry that recombinant human NCCRP1 is expressed intracellularly. Jaso-Friedmann, Leary and Evans previously reported two contradictory predictions of membrane topology and transmembrane helix location [7], [8]. Both papers suggest unrealistically short and fairly hydrophilic transmembrane helices but provide no explanation as to how these predictions were made. Moreover, both predictions locate the transmembrane regions in the middle of what we now know to be the beta sandwich fold of the FBA domain.

We speculate that the initial NCCRP1 research [7] was hampered by an unfortunate incident of purifying and cloning an incorrect protein with approximately the correct molecular weight (27.3 kDa) instead of the real antigen they originally discovered (34 kDa). In any case, our study now clearly demonstrates that NCCRP1 is an intracellular ubiquitin ligase protein, not a cell surface protein.

Previous reports provide some indirect results that have been interpreted as linking fish NCCRP1 proteins with cytotoxicity. First, *NCCRP1* anti-sense oligonucleotides were found to reduce the cytotoxicity of catfish NCC cells [7]. In our study, we have demonstrated the anti-proliferative effect of siRNA-mediated *NCCRP1* knockdown in HeLa cells, which may be due to a failure to degrade misfolded glycoproteins. We hypothesize that similar disturbances in viability or in protein secretion could account for the effect of anti-sense oligonucleotides in catfish NCC cells. Second, peptides derived from catfish and zebrafish NCCRP1 were observed to reduce NCC-mediated cell lysis [8], [12]. These peptides (Figs. 1 and 4) come from the most highly conserved region of the lectin domain and contain amphiphilic beta strands. We believe that the hydrophobic patches in these peptides would have a high degree of nonspecific binding and could interfere with various cell surface events. The uniform orientation of hydrophobic residues is sequence-dependent and would be lost in most randomized peptides, which may have been the case in a previous report [12], leading to seemingly specific results for a peptide that is not derived from a cell-surface receptor.

The human lectin-type F-box-only (FBXO) genes are present in two clusters in the genome: *FBXO2*, *FBXO6* and *FBXO44* on chromosome 1, and *FBXO17* and *FBXO27* on chromosome 19. *NCCRP1* is only 150 kb away from *FBXO27*, separated by two other genes. The adjacent gene triplet on human chromosome 1 and the pair on chromosome 19 are likely due to recent gene duplication events. However, the phylogenetic tree in Fig. 2B indicates that *FBXO17* and *FBXO27* share their latest common ancestor with the triplet of *FBXO* genes on chromosome 1, and therefore, their proximity to *NCCRP1* may be coincidental. A full phylogenetic analysis of FBXO proteins and their emergence in various vertebrate taxa would be an interesting topic for a future study.

Because there have been no previous reports on the expression pattern of *NCCRP1* in mammals, we examined mRNA levels of human and mouse *NCCRP1*. In normal human tissues, *NCCRP1* is strongly confined to the squamous epithelium, mainly being detected in tissues such as the esophagus, oral cavity, skin and tongue. The expression pattern is similar in cancerous tissues; the highest expression was seen in squamous cell carcinomas of the skin, cervix and vagina/vulva. In mice, the expression of *Nccrp1* was highest in the kidney and moderate in the stomach, colon, duodenum and prostate. Lower levels of the transcript were also detectable in the other tissues studied, including the parotid and submandibular glands, lung, jejunum, ileum and testis. These data suggest that NCCRP1 is relatively ubiquitously expressed, similar to FBXO6, FBXO27 and FBXO44 [15]. In contrast, FBXO2 is predominantly expressed in different areas of the brain and only weakly in some other tissues [15], [18], and FBXO17 is expressed in only a few tissues [15], [18]. It should be noted that many tissues express several FBXO genes. For example, major glycoprotein-producing tissues include the brain, liver and pancreas, all of which express multiple FBXO family members [15]. In this respect, the expression pattern of *NCCRP1* in human tissues is exceptional because it is almost solely present in tissues containing squamous epithelium. On the other hand, *Nccrp1* is expressed widely in mouse tissues; this pattern resembles the expression of other ubiquitously expressed FBXO genes.

Finally, we examined the connection between *CA9* and *NCCRP1* using several different approaches. *Nccrp1* was originally found to be significantly upregulated in the gastric mucosa of CA IX-deficient mice [6], which prompted us to explore its role more closely, but focusing on the human gene. In HeLa cells, we found that the NCCRP1 and CA IX proteins usually “avoid” each other; that is, they are only rarely expressed in the same cells (Fig. 8A, arrows). Furthermore, in a panel of 16 pancreatic cancer cell lines and 21 breast cancer cell lines, *NCCRP1* and *CA9* expression were inversely correlated in certain cell lines; *NCCRP1* was weakly expressed or absent when *CA9* mRNA was highly expressed, and vice versa. These data support the observation in *Car9* knockout mice in which the expression of *Nccrp1* was increased in the absence of *Car9*. We also determined whether the expression of *NCCRP1* was regulated in a similar manner to *CA9* in HeLa cells. *NCCRP1* expression decreased moderately after stimulation with deferoxamine mesylate, which was used to induce a hypoxia-like response. On the contrary, *CA9* expression increased highly, as expected. Thus, NCCRP1 does not seem to share a main regulatory pathway with CA IX.

siRNA-mediated gene silencing was used to study whether *CA9* expression directly regulates *NCCRP1* expression. Knockdown of *CA9* in U373 cells had no effect on *NCCRP1* expression levels, implying that *NCCRP1* is not directly regulated by *CA9*, at least in this cell line. Likewise, silencing of *NCCRP1* in HeLa and Su.86.86 cells did not affect *CA9* expression. Interestingly, we found that knockdown of *NCCRP1* led to a statistically significant decrease in the growth of HeLa cells, and this effect was still observed at 144 h after transfection. FBXO6 has also been shown to promote growth and proliferation of gastric cancer cells compared with control cells that do not express the protein [19]. One plausible explanation for these observations is that NCCRP1 and FBXO6 are needed to target certain glycoproteins that affect the cell cycle for degradation and that the proliferation of cancer cells is impaired when this function is inhibited. The results of this study indicate that *NCCRP1* is not directly regulated by *CA9*, but there are more complicated regulation events that lead to its upregulation when *CA9* is knocked down.

In conclusion, these studies provide ample evidence that the present name, “non-specific cytotoxic cell receptor protein 1,” does not fittingly describe the polypeptide known as NCCRP1. Bioinformatic analyses clearly show that NCCRP1 is a paralog to five other FBXO genes. We have also shown experimentally that the human recombinant NCCRP1 protein is expressed in the cytosol, not on the cell surface. Furthermore, the tissue expression pattern of NCCRP1 in humans and mice is incompatible with an immune receptor function.

## Materials and Methods

### Bioinformatics

Sequences were retrieved from Ensembl release 62 (www.ensembl.org) [20], UniProt (www.uniprot.org) [21], GenBank (www.ncbi.nlm.nih.gov/genbank/) [22] and RefSeq (www.ncbi.nlm.nih.gov/RefSeq/) [23]. BLAST searches were carried out via NCBI (blast.ncbi.nlm.nih.gov/Blast.cgi) [24]. Multiple sequence alignments were prepared with ClustalW (www.ebi.ac.uk/Tools/msa/clustalw2/) [25] and Mafft (www.ebi.ac.uk/Tools/msa/mafft/) [26] and visualized with GeneDoc (www.nrbsc.org/gfx/genedoc/). Protein motifs were searched using InterProScan (www.ebi.ac.uk/Tools/pfa/iprscan/) [27] and transmembrane domains were predicted with TMHMM v. 2.0 (www.cbs.dtu.dk/services/TMHMM/) [28]. Signal peptides and other target peptides were predicted using SignalP 3.0 with eukaryotic parameters (www.cbs.dtu.dk/services/SignalP/) [28] and TargetP 1.1. with non-plant parameters (www.cbs.dtu.dk/services/TargetP/) [28], respectively. Intrinsic protein disorder was predicted with DISpro (www.ics.uci.edu/~baldig/dispro.html) [29]. Phylogenetic trees were prepared using the MEGA4 software (www.megasoftware.net/) [30] with maximum parsimony and the complete deletion option for gapped sites with 1000 bootstrap replicates. Molecular images were prepared with PyMol 0.99 (DeLano Scientific, South San Francisco, CA).

### Construction of recombinant human NCCRP1

The full-length protein-coding cDNA sequence for human NCCRP1 was obtained from the Mammalian Gene Collection, and the cDNA clone (IMAGE ID: 30348184) was purchased from Source BioScience LifeSciences (Cambridge, United Kingdom). Plasmid DNA was isolated from an overnight culture using a QIAprep Spin Miniprep Kit (Qiagen, Hilden, Germany). *NCCRP1* cDNA was amplified by PCR using Phusion™ Hot Start High Fidelity DNA Polymerase (Finnzymes, Espoo, Finland). Primers were ordered from Biomers (Ulm, Germany). The forward primer sequence was 5′-CCG CGG ATC CAT GGA GGA GGT GCG TGA GGG A-3′ and the reverse primer was 5′-CGC CGT CGA CTC ACT CCC GGA GCT GCA CAG-3′, with the restriction sites for BamHI and SalI underlined, respectively. PCR was performed in an XP Thermal Cycler (Bioer Technology, Hangzhou, China) with a program consisting of a single 98°C denaturation step for 30 s followed by 35 cycles of denaturation at 98°C for 10 s, annealing at 64°C for 30 s and extension at 72°C for 30 s, and a final extension at 72°C for 5 min. The PCR product band was separated from agarose gel and dissolved using an Illustra™ GFX PCR DNA and GEL Band Purification Kit (GE Healthcare Life Sciences, Buckinghamshire, UK). The purified PCR product and pGEX-4T-1 vector (Invitrogen, Carlsbad, CA) were digested at 37°C for 2 hours with the BamHI and SalI restriction enzymes (New England Biolabs, Ipswich, MA, USA). The digested plasmid and NCCRP1 construct were purified and ligated overnight at 4°C using T4 DNA ligase (New England Biolabs). The ligated product was transformed into *E. coli* BL21(DE3)pLysS bacteria (Promega, Madison, WI, USA). Overnight cultures (5 ml) were grown from colonies, and plasmids were purified using a QIAprep Spin Miniprep Kit™ (Qiagen). Sequencing was performed to verify the sequence of the NCCRP1 construct. This purification method was based on a glutathione S-transferase (GST) gene fusion system. Expression of pGEX-4T-1 in *E. coli* yielded fusion proteins with the GST moiety at the amino terminus and NCCRP1 recombinant protein at the carboxyl terminus. The construct also contained a thrombin protease site between the GST tag and NCCRP1. This vector construct codes additional Gly and Ser residues to the N-terminus of the recombinant protein.

### Production and purification of recombinant human NCCRP1

A single BL21(DE3)pLysS transformant colony was grown in 5 ml LB medium containing 50 µg/ml ampicillin at room temperature with shaking (200 rpm) overnight and used to inoculate 500 ml of LB/amp medium. This culture was grown until the optical density (OD) at 600 nm reached 0.6. Expression of NCCRP1 protein was induced by adding isopropyl β-D-1-thiogalactopyranoside (IPTG) (Fermentas, Ontario, Canada) at a final concentration of 0.25 mM, and the culture was grown at room temperature overnight. The cells were harvested by centrifugation (Sorvall RC 28S) at 5,000 rpm for 5 min at room temperature. The cell pellet was suspended in 10 ml Tris-buffer containing 0.1 M Tris-Cl, pH 8, 0.05% Triton X-100, 200 mg lysozyme, 200 U DNase (Roche, Penzberg, Germany) and the protease inhibitors phenylmethanesulfonylfluoride (0.2 mg; Sigma-Aldrich, Helsinki, Finland) and leupeptin (0.1 mg) (Santa Cruz, Heidelberg, Germany). After a 30-min incubation at room temperature, the cell suspension was placed on ice and sonicated for 1 min. The suspension was clarified by centrifugation at 10,000 rpm for 30 min at 4°C, and the clear supernatant was affinity purified using Glutathione Sepharose 4B medium (GE Healthcare, Buckinghamshire, UK). Recombinant NCCRP1 protein was isolated under native conditions according to the manufacturer's protocol. A site-specific thrombin (GE Healthcare) was used for specific cleavage of the GST under shaking at room temperature overnight. Finally, the protein was eluted using the glutathione elution method (GE Healthcare) recommended by the manufacturer. The size of the expressed NCCRP1 protein was determined under reducing conditions by sodium dodecyl sulfate-polyacrylamide gel electrophoresis (SDS-PAGE) analysis.

### Production of a polyclonal antibody and western blotting

Anti-human NCCRP1 serum was raised in a rabbit against the purified full-length NCCRP1 by Innovagen AB (Lund, Sweden). Western blotting was used to confirm the reactivity of the antibody with NCCRP1.

Recombinant NCCRP1 proteins were subjected to SDS-PAGE under reducing conditions and transferred to polyvinylidene fluoride (PVDF) membranes. The membranes were treated with blocking solution (Santa Cruz, Heidelberg, Germany) for 30 min. After blocking, the membrane was incubated with the primary antibody (rabbit anti-NCCRP1) diluted 1∶2,000, or normal rabbit serum as a control, and washed. The membrane was incubated with the secondary antibody diluted 1∶25,000 (anti-rabbit Ig, horseradish-peroxidase-linked whole antibody from donkey, Amersham Biosciences, Buckinghamshire, England) and washed, and protein bands were visualized by electrochemiluminescence (ECL) using Amersham ECL™ Western Blotting Detection Reagents (GE Healthcare) according to the manufacturer's instructions.

### Mass spectrometry

All experiments were performed on a 4.7-T Fourier transform ion cyclotron resonance (FT-ICR) mass spectrometer (APEX-Qe; Bruker Daltonics, Billerica, MA, USA) equipped with an Apollo-II ion source and a mass-selective quadrupole front-end. This instrument has been described in detail elsewhere [31]. The protein samples were buffer exchanged into 10 mM ammonium acetate buffer, pH 6.9, on PD-10 columns (Amersham Biosciences, Billingham, UK) and directly electrosprayed at a flow rate of 1.5 µL/min. ESI-generated ions were externally accumulated for 1 s in a hexapole ion trap and transmitted to the ICR for trapping, excitation and detection. For each spectrum, 1,000 co-added 512-kWord time-domain transients were recorded, zero-filled twice, Gaussian multiplied and fast Fourier transformed before magnitude calculation and external mass calibration with respect to the ions of an ES Tuning Mix (Agilent Technologies, Santa Clara, CA, USA). All data were acquired and processed using Bruker XMASS 6.0.2 software. Mass spectra were further charge deconvoluted using a standard deconvolution macro implemented in the XMASS software. Tryptic digests were obtained by dissolving a small amount of the protein precipitate in 100 µl of a 10 mM ammonium bicarbonate buffer, pH 8.5, and 15 µg of sequencing grade trypsin (Promega GmbH, Mannheim, Germany) in 15 µl of water was added. The digest sample was incubated at 37°C for 1.5 hours, after which no precipitate was observed, and the sample was directly analyzed without chromatographic separation. The resulting protein or peptide masses were matched against the protein sequence using the GPMAW 8.11 software, and tryptic peptide masses were further subjected to database search using the Mascot search engine (www.matrixscience.com).

### Cell lines

A panel of 16 established pancreatic cancer cell lines was used in this study. Thirteen of these (AsPC-1, BxPC-3, Capan-1, Capan-2, CFPAC-1, HPAC, HPAF-II, Hs 700T, Hs 766T, MIA PaCa-2, PANC-1, Su.86.86, and SW 1990) were purchased from the American Type Culture Collection (ATCC, Manassas, VA, USA), and three (DanG, Hup-T3, and Hup-T4) were purchased from the German Collection of Microorganisms and Cell Cultures (Braunschweig, Germany). The 21 breast cancer cell lines used in this study (BT-474, CAMA-1, DU4475, HCC38, HCC1419, HCC1954, MCF7,MDA-MB-134-VI, MDA-MB-231, MDA-MB-361, MDA-MB-415, MDA-MB-436, MDA-MB-453, SK-BR-3, T-47D, UACC-732, UACC-812, UACC-893, UACC-3133, ZR-75-1, and ZR-75-30) were obtained from the ATCC. The U373 human glioblastoma astrocytoma cell line was purchased from the European Collection of Cell Cultures. The HeLa human cervical carcinoma cell line was from the laboratory of Professor Jorma Isola (Institute of Biomedical Technology, University of Tampere, Tampere, Finland). Cells were grown under the recommended culture conditions.

### Immunocytochemistry

Rabbit anti-human NCCRP1 serum (Innovagen) and the M75 antibody specific for the PG region of CA IX were used for immunocytochemistry [32]. Non-immune normal rabbit serum was used as a control.

HeLa cells were fixed with 4% (vol/vol) neutral-buffered formaldehyde for 30 min. The cells were then rinsed with PBS and subjected to immunofluorescence staining using the following protocol: (*a*) pre-treatment with 0.1% BSA in PBS (BSA-PBS) for 30 min; (*b*) incubation for 1 h with rabbit NCCRP1 antiserum or normal rabbit serum diluted 1∶100 in 0.1% BSA-PBS or mouse CA IX antiserum diluted 1∶10 in 0.1% BSA-PBS; (*c*) rinsing three times for 5 min with BSA-PBS; (*d*) incubation for 1 h with 1∶100 diluted Alexa Fluor 488 goat anti-rabbit IgG antibodies or Alexa Fluor 568 goat anti-mouse IgG antibodies (both from Molecular Probes, Eugene, Oregon, USA) in 0.1% BSA-PBS; (*e*) rinsing two times for 5 min with BSA-PBS and once with PBS. All incubations and washings were performed in the presence of 0.05% saponin. Immunostained cells were analyzed and photographed using a Zeiss LSM 700 confocal laser scanning microscope.

### *NCCRP1* expression in human and mouse tissues

*NCCRP1* mRNA expression levels across a large number of human tissues were retrieved from the IST database system developed by MediSapiens Ltd. The current version of the IST database (4.3) contains 20,218 human tissue and cell line samples analyzed by Affymetrix gene expression microarrays. The database was constructed and validated using methods similar to those described by Kilpinen et al. [33] and Autio et al. [34] for the construction of the GeneSapiens database. The database contains expression data that is fully integrated in terms of both the annotation and numerical compatibility, allowing for analysis of the expression levels of 19,123 genes across 228 distinct tissues.

Mouse tissues were obtained from C57BL/6 mice that were maintained in the animal facility of the University of Oulu. A total of six mice (3 males and 3 females) were sacrificed at two months of age. Tissue specimens were taken from the following areas: parotid gland, submandibular gland, stomach, duodenum, jejunum, ileum, colon, lung, kidney, prostate and testis. The tissue samples were immediately immersed in RNAlater solution (Ambion, Austin, TX, USA) and frozen at −80°C.

Total RNA was extracted from the mouse tissues using an RNeasy RNA isolation kit (Qiagen, Valencia, USA) following the manufacturer's instructions. Residual DNA was removed from the samples using RNase-free DNase (Qiagen). The RNA concentration and purity was determined by measuring the optical density at 260 and 280 nm. Different quantities of RNA (lung and prostate: 200 ng, ileum: 900 ng, all other tissues: 2,000 ng) were converted into first strand cDNA using a First Strand cDNA synthesis kit (Fermentas, Burlington, Canada) with random hexamer primers according to the manufacturer's protocol.

The relative expression levels of the mouse *Nccrp1* gene in several tissues were assessed by quantitative real-time PCR (QRT-PCR) using the LightCycler detection system (Roche, Rotkreuz, Switzerland). The primers (Table 2) were designed using Primer3 (http://frodo.wi.mit.edu/primer3/) and based on the complete cDNA sequence deposited in GenBank. The specificity of the primers was verified using NCBI Basic Local Alignment Search Tool (BLAST) (http://blast.ncbi.nlm.nih.gov/Blast.cgi). The housekeeping gene *β-actin* (*Actb*) was used as internal control to normalize the cDNA samples for possible differences in quality and quantity (Table 2).

**Table 2:** QRT-PCR primer sequences used in this study.

Gene symbol	Description	GenBank number	Forward primer (5′-3′)	Reverse primer (5′-3′)
*Nccrp1*	Mus musculus non-specific cytotoxic cell receptor protein 1 homolog(zebrafish)	NM_001081115	GCTGCATGTCTGGCTGTTAG	ATGCGGTTCTTAGCCTTGTG
*Actb*	Mus musculus actin, beta	NM_007393	AGAGGGAAATCGTGCGTGAC	CAATAGTGATGACCTGGCCGT
*NCCRP1*	Homo sapiens non-specific cytotoxic cell receptor protein 1 homolog(zebrafish)	NM_001001414	TTCCGTGGCTGGTACATTAG	ATGGCTGGTTGTTCGTCATC
*CA9*	Homo sapiens carbonic anhydrase IX	NM_001216	ATGAGAAGGCAGCACAGAAG	TAATGAGCAGGACAGGACAG
*UBC*	Homo sapiens ubiquitin C	NM_021009	ATTTGGGTCGCGGTTCTTG	TGCCTTGACATTCTCGATGGT
*GAPDH*	Homo sapiens glyceraldehyde-3-phosphate dehydrogenase	NM_002046	TGCACCACCAACTGCTTAGC	GGCATGGACTGTGGTCATGAG

Each PCR reaction was performed in a total volume of 20 µl containing 1.0 µl of first strand cDNA, 1× QuantiTect SYBR Green PCR Master Mix (Qiagen, Hilden, Germany) and 0.5 µM of each primer. Amplification and detection were conducted as follows: after an initial 15 min activation step at 95°C, amplification was performed in a three-step cycling procedure for 45 cycles consisting of denaturation at 95°C for 15 s, annealing at a temperature determined according to the T_m_ for each primer pair for 20 s, and elongation at 72°C for 15 s (the ramp rate was 20°C/s for all the steps), and a final cooling step was performed. Melting curve analysis was always performed after the amplification to check the specificity of the PCR reaction. To quantify the levels of transcripts in the cell lines studied, a standard curve was established for each gene using five-fold serial dilutions of known concentrations of purified PCR products generated with the same primer pairs. Each cDNA sample was tested in triplicate, and the crossing point (Cp) value obtained allowed the levels of the starting mRNA to be determined using a specific standard curve. The relative mRNA expression was calculated as the copy number of the target gene divided by the corresponding normalization factor and multiplied by 10^3^. Median values were calculated from the technical triplicates.

### The effect of growth factors and deferoxamine mesylate on mRNA expression of *NCCRP1*

HeLa cells were grown in 75 cm^2^ flasks in a 37°C incubator with humidified 5% CO_2_/95% air. When the cultured cells reached 80–90% confluence, they were trypsinized and plated in 58 cm^2^ dishes at a density of 1 million cells per dish. After 24 hours, the medium was replaced with fresh medium containing recombinant human transforming growth factor-alpha (TGF-α; 10 ng/ml), recombinant human transforming growth factor-beta 1 (TGF-β1; 10 ng/ml), recombinant human epidermal growth factor (EGF; 10 ng/ml), or deferoxamine mesylate (200 µM), an iron chelator commonly used to induce the hypoxia regulatory pathway. Normal medium was added to control plates. TGF-β1 and EGF were purchased from ProSpec-Tany TechnoGene Ltd. (Rehovot, Israel). TGF-α was purchased from PromoCell GmbH (Heidelberg, Germany). Deferoxamine mesylate was obtained from Sigma-Aldrich Finland Oy (Helsinki, Finland). The chemicals were diluted as necessary according to the manufacturers' instructions. The cells were incubated for 72 hours, after which they were harvested and total RNA was extracted using an RNeasy RNA isolation kit. For each sample, 850 ng of RNA was converted into first strand cDNA by reverse transcription and QRT-PCR was performed as described above. The primers for human *NCCRP1* and *CA9* were designed using Primer3 and the primers for the housekeeping gene ubiquitin C (*UBC*) were obtained from the RTprimerDB database (http://www.rtprimerdb.org/) under the identification number 8 (Table 2). The median values were calculated from the technical triplicates for the QRT-PCR experiments. Subsequently, the median values for treatments were compared to the median values for negative controls.

### Expression of *CA9* and *NCCRP1* in human pancreatic and breast cancer cell lines

To examine the co-expression of human *CA9* and *NCCRP1* in tumor cells, transcript levels were measured in 16 pancreatic and 21 breast cancer cell lines using QRT-PCR. Total RNA was isolated from the cell lines using TRIzol reagent (Invitrogen, Carlsbad, CA), and 5 µg of each RNA sample was converted into first strand cDNA using SuperScript III First-Strand Synthesis kit (Invitrogen) according to the manufacturer's instructions. QRT-PCR reactions were performed as described above, with the exception that each cDNA sample was tested only once. The housekeeping gene glyceraldehyde-3-phosphate dehydrogenase (*GAPDH*) was used as a reference (Table 2). The final relative mRNA expression was calculated as the copy number of the target gene divided by the corresponding normalization factor, multiplied by 10^4^.

### *NCCRP1* and *CA9* silencing

The human *NCCRP1* and *CA9* genes were silenced using specific ON-TARGETplus SMARTpool small interfering RNAs (siRNAs) (Thermo Fisher Scientific, Lafayette, CO). The catalog numbers for *NCCRP1* and *CA9* SMARTpools are L-032307-01-0005 and L-005244-00-0005, respectively. U373 cells, HeLa cells and Su.86.86 cells were transfected with the siRNAs using INTERFERin™ reagent (PolyPlus-transfection, Illkirch, France) according to the manufacturer's instructions. Transfections were performed in 24-well plates with the desired cell density (25,000 cells per well for U373 and HeLa cells and 50,000 cells per well for Su.86.86 cells). Final concentrations of 10 nM for *CA9* siRNA and 30 nM for *NCCRP1* siRNA were used. Parallel control experiments using siRNA targeting the firefly *luciferase* (*PPYLUC*) gene were also performed. The sequence of the *PPYLUC* siRNA was 5′-GAUUUCGAGUCGUCUUAAUTT-3′. All siRNA experiments were performed in triplicate and repeated twice. Gene silencing was verified each time using QRT-PCR as described above. The housekeeping gene glyceraldehyde-3-phosphate dehydrogenase (*GAPDH*) was used as a reference (Table 2). Median values were calculated from the triplicate QRT-PCR experiments, and the median values for *NCCRP1* or *CA9* siRNA-treated cells were compared to the median values for *luciferase* control siRNA-treated cells to estimate the efficacy of silencing.

### Cell growth analyses

For cell growth analyses, 15,000 HeLa cells per well were transfected with *NCCRP1* SMARTpool siRNAs or *luciferase* control siRNA as described above. Cell growth was analyzed at 48, 96, and 144 hours after transfection.

From each well, 4×4 fields were photographed using Olympus IX71 microscope with a 100× objective. Quantitative analysis of the captured images was performed using the public domain image processing software ImageJ (http://rsbweb.nih.gov/ij/). A custom-made algorithm was developed for the analysis; it first identified the cellular area of the sample image at each time point and then measured the relative change in the cellular area percentage over the sample-specific time course.

### Statistical analyses

For the cell growth analyses, the Mann-Whitney test was used to evaluate differences in group values for *NCCRP1* SMARTpool siRNA-treated HeLa cells vs. *luciferase* control siRNA-treated HeLa cells.

### Ethics statement

The animal protocols were approved by the Animal Care and Use Committee of the University of Oulu (Permit Number: 085/07).

## Supporting Information

Figure S1 **A search result against the SwissProt database for trypsin digestion of recombinant human NCCRP1.** A search against the SwissProt database gave an unambiguous hit for human NCCRP1 (NCRP1_HUMAN) with a Mowse score of 370.
(PDF)
