# Supplementary material for: Characterization of Non-Specific Cytotoxic Cell Receptor Protein 1: A New Member of the Lectin-Type Subfamily of F-Box Proteins
Source: PLoS One. 2011 Nov 7;6(11):e27152. doi: 10.1371/journal.pone.0027152 (PMC3210139; doi:10.1371/journal.pone.0027152)
Supplement: Figure S1 — A search result against the SwissProt database for trypsin digestion of recombinant human NCCRP1. A search against the SwissProt database gave an unambiguous hit for human NCCRP1 (NCRP1_HUMAN) with a Mowse score of 370. (PDF) [file pone.0027152.s001.pdf]

{MATRIX SCIENCE} Mascot Search Results

Protein View

Match to: **NCRP1_HUMAN** Score: **370** Expect: **5.3e-32**
Non-specific cytotoxic cell receptor protein 1 homolog OS=Homo sapiens GN=NCCRP1 PE=1 SV=1

Nominal mass (M_r): **30828**; Calculated pI value: **6.16**
NCBI BLAST search of NCRP1_HUMAN against nr
Unformatted sequence string for pasting into other applications

Taxonomy: Homo sapiens

Cleavage by Trypsin: cuts C-term side of KR unless next residue is P
Number of mass values searched: **21**
Number of mass values matched: **21**
Sequence Coverage: **75%**

Matched peptides shown in **Bold Red**

```
  1 MEEVREGHAL GGGMEADGPA SLQELPPSPR SPSPPPSPPP LPSPPSLPSP
 51 AAPEAPELPE PAQPSEAHAR QLLLEEWGPL SGGLELPQRL TWKLLLLRRP
101 LYRNLLRSPN PEGINIYEPA PPTGPTQRPL ETLGNFRGWY IRTEKLQQNQ
151 SWTVKQQCVD LLAEGLWEEL LDDEQPAITV MDWFEDSRLD ACVYELHVWL
201 LAADRRTVIA QHHVAPRTSG RGPPGRWVQV SHVFRHYGPG VRFIHFLHKA
251 KNRMEPGGLR RTRVTDSSVS VQLRE
```

Show predicted peptides also

Sort Peptides By Residue Number Increasing Mass Decreasing Mass

Start - End	Observed	Mr (expt)	Mr (calc)	ppm	Miss	Sequence
6 - 30	2472.1823	2472.1823	2472.1703	5	0	R.EGHALGGGMEADGPASLQELPPSPR.S
31 - 70	3980.0149	3980.0149	3979.9955	5	0	R.SPSPPPSPPPLPSPPSLPSPAAPEAPELPEPAQPSEAHAR.Q
31 - 70	3980.0213	3980.0213	3979.9955	6	0	R.SPSPPPSPPPLPSPPSLPSPAAPEAPELPEPAQPSEAHAR.Q
71 - 89	2134.1533	2134.1533	2134.1422	5	0	R.QLLLEEWGPLSGGLELPQR.L
90 - 93	546.3196	546.3196	546.3166	6	0	R.LTWK.L
94 - 98	626.4510	626.4510	626.4479	5	0	K.LLLLR.R
99 - 103	703.4167	703.4167	703.4129	5	0	R.RPLYR.N
104 - 107	514.3256	514.3256	514.3227	6	0	R.NLLR.S
108 - 137	3261.6589	3261.6589	3261.6418	5	0	R.SPNPEGINIYEPAPPTGPTQRPLETLGNFR.G
108 - 137	3261.6695	3261.6695	3261.6418	8	0	R.SPNPEGINIYEPAPPTGPTQRPLETLGNFR.G
138 - 142	693.3636	693.3636	693.3598	5	0	R.GWYIR.T
146 - 155	1230.6425	1230.6425	1230.6357	6	0	K.LQQNQSWTVK.Q
207 - 217	1227.6899	1227.6899	1227.6836	5	0	R.TVIAQHHVAPR.T
207 - 217	1227.6912	1227.6912	1227.6836	6	0	R.TVIAQHHVAPR.T
218 - 221	419.2148	419.2148	419.2128	5	0	R.TSGR.G
222 - 226	482.2686	482.2686	482.2601	18	0	R.GPPGR.W
227 - 235	1156.6199	1156.6199	1156.6142	5	0	R.WVQVSHVFR.H
236 - 242	784.4027	784.4027	784.3980	6	0	R.HYGPGVR.F
243 - 249	940.5332	940.5332	940.5283	5	0	R.FIHFLHK.A
254 - 260	758.3787	758.3787	758.3745	6	0	R.MEPGGLR.R
264 - 274	1189.6367	1189.6367	1189.6303	5	0	R.VTDSSVSVQLR.E

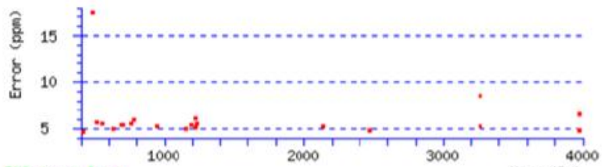

RMS error 6 ppm

```
AC   Q6ZVX7; Q6NVV5;
DT   18-MAR-2008, integrated into UniProtKB/Swiss-Prot.
DT   05-JUL-2004, sequence version 1.
DT   05-APR-2011, entry version 57.
DE   RecName: Full=Non-specific cytotoxic cell receptor protein 1 homolog;
GN   Name=NCCRP1;
OS   Homo sapiens (Human).
OC   Eukaryota; Metazoa; Chordata; Craniata; Vertebrata; Euteleostomi;
OC   Mammalia; Eutheria; Euarchontoglires; Primates; Haplorrhini;
OC   Catarrhini; Hominidae; Homo.
OX   NCBI_TaxID=9606;
RN   [1]
RP   NUCLEOTIDE SEQUENCE [LARGE SCALE MRNA].
```

```
RC   TISSUE=Placenta;
RX   PubMed=14702039; DOI=10.1038/ng1285;
RA   Ota T., Suzuki Y., Nishikawa T., Otsuki T., Sugiyama T., Irie R.,
RA   Wakamatsu A., Hayashi K., Sato H., Nagai K., Kimura K., Makita H.,
RA   Sekine M., Obayashi M., Nishi T., Shibahara T., Tanaka T., Ishii S.,
RA   Yamamoto J., Saito K., Kawai Y., Isono Y., Nakamura Y., Nagahari K.,
RA   Murakami K., Yasuda T., Iwayanagi T., Wagatsuma M., Shiratori A.,
RA   Sudo H., Hosoiri T., Kaku Y., Kodaira H., Kondo H., Sugawara M.,
RA   Takahashi M., Kanda K., Yokoi T., Furuya T., Kikkawa E., Omura Y.,
RA   Abe K., Kamihara K., Katsuta N., Sato K., Tanikawa M., Yamazaki M.,
RA   Ninomiya K., Ishibashi T., Yamashita H., Murakawa K., Fujimori K.,
RA   Tanai H., Kimata M., Watanabe M., Hiraoka S., Chiba Y., Ishida S.,
RA   Ono Y., Takiguchi S., Watanabe S., Yosida M., Hotuta T., Kusano J.,
RA   Kanehori K., Takahashi-Fujii A., Hara H., Tanase T.-O., Nomura Y.,
RA   Togiya S., Komai F., Hara R., Takeuchi K., Arita M., Imose N.,
RA   Musashino K., Yuuki H., Oshima A., Sasaki N., Aotsuka S.,
RA   Yoshikawa Y., Matsunawa H., Ichihara T., Shiohata N., Sano S.,
RA   Moriya S., Momiyama H., Satoh N., Takami S., Terashima Y., Suzuki O.,
RA   Nakagawa S., Senoh A., Mizoguchi H., Goto Y., Shimizu F., Wakebe H.,
RA   Hishigaki H., Watanabe T., Sugiyama A., Takemoto M., Kawakami B.,
RA   Yamazaki M., Watanabe K., Kumagai A., Itakura S., Fukuzumi Y.,
RA   Fujimori Y., Komiyama M., Tashiro H., Tanigami A., Fujiwara T.,
RA   Ono T., Yamada K., Fujii Y., Ozaki K., Hirao M., Ohmori Y.,
RA   Kawabata A., Hikiji T., Kobatake N., Inagaki H., Ikema Y., Okamoto S.,
RA   Okitani R., Kawakami T., Noguchi S., Itoh T., Shigeta K., Senba T.,
RA   Matsumura K., Nakajima Y., Mizuno T., Morinaga M., Sasaki M.,
RA   Togashi T., Oyama M., Hata H., Watanabe M., Komatsu T.,
RA   Mizushima-Sugano J., Satoh T., Shirai Y., Takahashi Y., Nakagawa K.,
RA   Okumura K., Nagase T., Nomura N., Kikuchi H., Masuho Y., Yamashita R.,
RA   Nakai K., Yada T., Nakamura Y., Ohara O., Isogai T., Sugano S.;
RT   "Complete sequencing and characterization of 21,243 full-length human
RT   cDNAs.";
RL   Nat. Genet. 36:40-45(2004).
RN   [2]
RP   NUCLEOTIDE SEQUENCE [LARGE SCALE MRNA].
RC   TISSUE=Placenta;
RX   PubMed=15489334; DOI=10.1101/gr.2596504;
RG   The MGC Project Team;
RT   "The status, quality, and expansion of the NIH full-length cDNA
RT   project: the Mammalian Gene Collection (MGC).";
RL   Genome Res. 14:2121-2127(2004).
CC   -!- SIMILARITY: Contains 1 FBA (F-box associated) domain.
CC   -----------------------------------------------------------------------
CC   Copyrighted by the UniProt Consortium, see http://www.uniprot.org/terms
CC   Distributed under the Creative Commons Attribution-NoDerivs License
CC   -----------------------------------------------------------------------
DR   EMBL; AK123941; BAC85732.1; -; mRNA.
DR   EMBL; BC067874; AAH67874.2; -; mRNA.
DR   EMBL; BC092493; AAH92493.1; -; mRNA.
DR   IPI; IPI00247167; -.
DR   RefSeq; NP_001001414.1; NM_001001414.1.
DR   UniGene; Hs.726934; -.
DR   HSSP; Q80UW2; 1UMH.
DR   ProteinModelPortal; Q6ZVX7; -.
DR   SMR; Q6ZVX7; 86-272.
DR   STRING; Q6ZVX7; -.
DR   PhosphoSite; Q6ZVX7; -.
DR   PeptideAtlas; Q6ZVX7; -.
DR   PRIDE; Q6ZVX7; -.
DR   Ensembl; ENST00000339852; ENSP00000342137; ENSG00000188505.
DR   GeneID; 342897; -.
DR   KEGG; hsa:342897; -.
DR   NMPDR; fig|9606.3.peg.16409; -.
DR   UCSC; uc002okq.1; human.
DR   CTD; 342897; -.
DR   GeneCards; GC19P036134; -.
DR   HGNC; HGNC:33739; NCCRP1.
DR   neXtProt; NX_Q6ZVX7; -.
DR   PharmGKB; PA164723566; -.
DR   eggNOG; prNOG16065; -.
DR   GeneTree; ENSGT00390000003865; -.
DR   HOGENOM; HBG716440; -.
DR   HOVERGEN; HBG099012; -.
DR   InParanoid; Q6ZVX7; -.
DR   OMA; FLHKAKN; -.
DR   OrthoDB; EOG4BCDP1; -.
DR   PhylomeDB; Q6ZVX7; -.
DR   NextBio; 98367; -.
DR   ArrayExpress; Q6ZVX7; -.
DR   Bgee; Q6ZVX7; -.
DR   CleanEx; HS_NCCRP1; -.
DR   Genevestigator; Q6ZVX7; -.
DR   GO; GO:0030163; P:protein catabolic process; IEA:InterPro.
```

```
DR   InterPro; IPR007397; F-box-assoc_dom.
DR   InterPro; IPR008979; Galactose-bd-like.
DR   Pfam; PF04300; FBA; 1.
DR   SUPFAM; SSF49785; Gal_bind_like; 1.
DR   PROSITE; PS51114; FBA; 1.
PE   1: Evidence at protein level;
KW   Complete proteome.
FT   CHAIN         1    275       Non-specific cytotoxic cell receptor
FT                                protein 1 homolog.
FT                                /FTId=PRO_0000326028.
FT   DOMAIN       95    273       FBA.
FT   COMPBIAS     26     64       Pro-rich.
SQ   SEQUENCE   275 AA;  30847 MW;  FB9C700E1799E50E CRC64;
     MEEVREGHAL GGGMEADGPA SLQELPPSPR SPSPPPSPPP LPSPPSLPSP AAPEAPELPE
     PAQPSEAHAR QLLLEEWGPL SGGLELPQRL TWKLLLLRRP LYRNLLRSPN PEGINIYEPA
     PPTGPTQRPL ETLGNFRGWY IRTEKLQQNQ SWTVKQQCVD LLAEGLWEEL LDDEQPAITV
     MDWFEDSRLD ACVYELHVWL LAADRRTVIA QHHVAPRTSG RGPPGRWVQV SHVFRHYGPG
     VRFIHFLHKA KNRMEPGGLR RTRVTDSSVS VQLRE
```

Mascot: http://www.matrixscience.com/

Mascot Search Results

**User**	: janne
**Email**	: janne.janis@uef.fi
**Search title**	: nccrp1
**Database**	: SwissProt 2011_04 (526969 sequences; 186402391 residues)
**Timestamp**	: 20 Apr 2011 at 12:14:15 GMT
**Warning**	: Specifying a protein mass disables mixture mode in PMF-search
**Top Score**	: 370 for NCRP1_HUMAN, Non-specific cytotoxic cell receptor protein 1 homolog OS=Homo sapiens GN=NCCRP1 PE=1 SV=1

Mascot Score Histogram

Protein score is -10*Log(P), where P is the probability that the observed match is a random event.
Protein scores greater than 70 are significant (p<0.05).

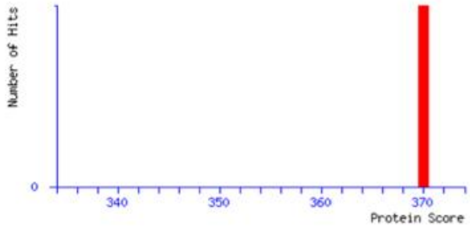

Concise Protein Summary Report

Format As Concise Protein Summary Help

Significance threshold p< 0.05 Max. number of hits AUTO

Re-Search All Search Unmatched

1. NCRP1_HUMAN **Mass:** 30828 **Score:** 370 **Expect:** 5.3e-32 **Matches:** 21
Non-specific cytotoxic cell receptor protein 1 homolog OS=Homo sapiens GN=NCCRP1 PE=1 SV=1
RK3_BIGNA **Mass:** 33104 **Score:** 46 **Expect:** 15 **Matches:** 5
50S ribosomal protein L3, chloroplastic OS=Bigelowiella natans GN=RPL3 PE=2 SV=1
YDEO_ECOLI **Mass:** 28707 **Score:** 39 **Expect:** 66 **Matches:** 5
HTH-type transcriptional regulator ydeO OS=Escherichia coli (strain K12) GN=ydeO PE=2 SV=1
YDEO_SHIFL **Mass:** 28778 **Score:** 39 **Expect:** 71 **Matches:** 5
HTH-type transcriptional regulator ydeO OS=Shigella flexneri GN=ydeO PE=2 SV=1
YDEO_ECOL6 **Mass:** 28930 **Score:** 39 **Expect:** 73 **Matches:** 5
HTH-type transcriptional regulator ydeO OS=Escherichia coli O6 GN=ydeO PE=2 SV=1
PYRE_HELAH **Mass:** 21962 **Score:** 37 **Expect:** 1.1e+02 **Matches:** 4
Orotate phosphoribosyltransferase OS=Helicobacter acinonychis (strain Sheeba) GN=pyrE PE=3 SV=1
DUT_EHV1B **Mass:** 35187 **Score:** 35 **Expect:** 1.6e+02 **Matches:** 4
Deoxyuridine 5'-triphosphate nucleotidohydrolase OS=Equine herpesvirus 1 (strain Ab4p) GN=9 PE=3 SV=1
DNLJ_METI4 **Mass:** 77370 **Score:** 35 **Expect:** 1.7e+02 **Matches:** 5
DNA ligase OS=Methylacidiphilum infernorum (isolate V4) GN=ligA PE=3 SV=1
GLMS_OCEIH **Mass:** 66184 **Score:** 35 **Expect:** 1.8e+02 **Matches:** 5
Glucosamine--fructose-6-phosphate aminotransferase [isomerizing] OS=Oceanobacillus iheyensis GN=glmS PE=3 SV=3
ARLY_BURP8 **Mass:** 51037 **Score:** 34 **Expect:** 2.4e+02 **Matches:** 5
Argininosuccinate lyase OS=Burkholderia phymatum (strain DSM 17167 / STM815) GN=argH PE=3 SV=1
MAGBI_MACFA **Mass:** 38582 **Score:** 33 **Expect:** 2.5e+02 **Matches:** 4
Melanoma-associated antigen B18 OS=Macaca fascicularis GN=MAGEB18 PE=2 SV=1
TAL_BURXL **Mass:** 34886 **Score:** 32 **Expect:** 3.2e+02 **Matches:** 4
Transaldolase OS=Burkholderia xenovorans (strain LB400) GN=tal PE=3 SV=1
RRMF_UREP2 **Mass:** 18437 **Score:** 32 **Expect:** 3.5e+02 **Matches:** 4
Probable rRNA maturation factor OS=Ureaplasma parvum serovar 3 (strain ATCC 27815 / 27 / NCTC 11736) GN=UPA3_0508 PE=3 SV=1
RRMF_UREPA **Mass:** 18437 **Score:** 32 **Expect:** 3.5e+02 **Matches:** 4
Probable rRNA maturation factor OS=Ureaplasma parvum GN=UU490 PE=3 SV=1
TPPC5_DICDI **Mass:** 20733 **Score:** 31 **Expect:** 3.9e+02 **Matches:** 3
Trafficking protein particle complex subunit 5 OS=Dictyostelium discoideum GN=trappc5 PE=3 SV=1
SUCC_RALSO **Mass:** 41149 **Score:** 31 **Expect:** 4.3e+02 **Matches:** 5
Succinyl-CoA ligase [ADP-forming] subunit beta OS=Ralstonia solanacearum GN=sucC PE=3 SV=1
CLPS_PSEA6 **Mass:** 12351 **Score:** 31 **Expect:** 4.4e+02 **Matches:** 3
ATP-dependent Clp protease adapter protein ClpS OS=Pseudoalteromonas atlantica (strain T6c / BAA-1087) GN=clpS PE=3 SV=1
PNCB_SALA4 **Mass:** 45673 **Score:** 31 **Expect:** 4.4e+02 **Matches:** 4
Nicotinate phosphoribosyltransferase OS=Salmonella agona (strain SL483) GN=pncB PE=3 SV=1
PNCB_SALCH **Mass:** 45705 **Score:** 31 **Expect:** 4.4e+02 **Matches:** 4
Nicotinate phosphoribosyltransferase OS=Salmonella choleraesuis GN=pncB PE=3 SV=2
PNCB_SALDC **Mass:** 45647 **Score:** 31 **Expect:** 4.4e+02 **Matches:** 4
Nicotinate phosphoribosyltransferase OS=Salmonella dublin (strain CT_02021853) GN=pncB PE=3 SV=1
PNCB_SALEP **Mass:** 45647 **Score:** 31 **Expect:** 4.4e+02 **Matches:** 4
Nicotinate phosphoribosyltransferase OS=Salmonella enteritidis PT4 (strain P125109) GN=pncB PE=3 SV=1
PNCB_SALG2 **Mass:** 45647 **Score:** 31 **Expect:** 4.4e+02 **Matches:** 4
Nicotinate phosphoribosyltransferase OS=Salmonella gallinarum (strain 287/91 / NCTC 13346) GN=pncB PE=3 SV=1

PNCB_SALNS **Mass:** 45678 **Score:** 31 **Expect:** 4.4e+02 **Matches:** 4
Nicotinate phosphoribosyltransferase OS=Salmonella newport (strain SL254) GN=pncB PE=3 SV=1
PNCB_SALPA **Mass:** 45678 **Score:** 31 **Expect:** 4.4e+02 **Matches:** 4
Nicotinate phosphoribosyltransferase OS=Salmonella paratyphi A GN=pncB PE=3 SV=1
PNCB_SALPB **Mass:** 45678 **Score:** 31 **Expect:** 4.4e+02 **Matches:** 4
Nicotinate phosphoribosyltransferase OS=Salmonella paratyphi B (strain ATCC BAA-1250 / SPB7) GN=pncB PE=3 SV=1
PNCB_SALPC **Mass:** 45647 **Score:** 31 **Expect:** 4.4e+02 **Matches:** 4
Nicotinate phosphoribosyltransferase OS=Salmonella paratyphi C (strain RKS4594) GN=pncB PE=3 SV=1
PNCB_SALPK **Mass:** 45678 **Score:** 31 **Expect:** 4.4e+02 **Matches:** 4
Nicotinate phosphoribosyltransferase OS=Salmonella paratyphi A (strain AKU_12601) GN=pncB PE=3 SV=1
PNCB_SALSV **Mass:** 45692 **Score:** 31 **Expect:** 4.4e+02 **Matches:** 4
Nicotinate phosphoribosyltransferase OS=Salmonella schwarzengrund (strain CVM19633) GN=pncB PE=3 SV=1
PNCB_SALTI **Mass:** 45664 **Score:** 31 **Expect:** 4.4e+02 **Matches:** 4
Nicotinate phosphoribosyltransferase OS=Salmonella typhi GN=pncB PE=3 SV=3
PNCB_SALTY **Mass:** 45632 **Score:** 31 **Expect:** 4.4e+02 **Matches:** 4
Nicotinate phosphoribosyltransferase OS=Salmonella typhimurium GN=pncB PE=3 SV=2
RBFA_MYCPN **Mass:** 13381 **Score:** 30 **Expect:** 4.7e+02 **Matches:** 3
Ribosome-binding factor A OS=Mycoplasma pneumoniae GN=rbfA PE=1 SV=1
PRTE_ERWCH **Mass:** 49549 **Score:** 30 **Expect:** 5.4e+02 **Matches:** 5
Proteases secretion protein prtE OS=Erwinia chrysanthemi GN=prtE PE=3 SV=1
SC23A_XENTR **Mass:** 85983 **Score:** 30 **Expect:** 5.5e+02 **Matches:** 4
Protein transport protein Sec23A OS=Xenopus tropicalis GN=sec23a PE=2 SV=1
PURA_AROAE **Mass:** 47037 **Score:** 30 **Expect:** 5.5e+02 **Matches:** 4
Adenylosuccinate synthetase OS=Aromatoleum aromaticum (strain EbN1) GN=purA PE=3 SV=1
Y609_MYCTU **Mass:** 14524 **Score:** 30 **Expect:** 5.8e+02 **Matches:** 3
UPF0110 protein Rv0609/MT0638 OS=Mycobacterium tuberculosis GN=Rv0609 PE=3 SV=1
Y625_MYCBO **Mass:** 14524 **Score:** 30 **Expect:** 5.8e+02 **Matches:** 3
UPF0110 protein Mb0625 OS=Mycobacterium bovis GN=Mb0625 PE=3 SV=1
DHQS_METM6 **Mass:** 39857 **Score:** 30 **Expect:** 5.9e+02 **Matches:** 4
3-dehydroquinate synthase OS=Methanococcus maripaludis (strain C6 / ATCC BAA-1332) GN=MmarC6_0943 PE=3 SV=1
ACPS_CHLT3 **Mass:** 13874 **Score:** 29 **Expect:** 6.2e+02 **Matches:** 3
Holo-[acyl-carrier-protein] synthase OS=Chloroherpeton thalassium (strain ATCC 35110 / GB-78) GN=acpS PE=3 SV=1
Y1683_STREM **Mass:** 40594 **Score:** 29 **Expect:** 6.2e+02 **Matches:** 4
UPF0348 protein Sez_1683 OS=Streptococcus equi subsp. zooepidemicus (strain MGCS10565) GN=Sez_1683 PE=3 SV=1
Y1906_STRE4 **Mass:** 40583 **Score:** 29 **Expect:** 6.2e+02 **Matches:** 4
UPF0348 protein SEQ_1906 OS=Streptococcus equi subsp. equi (strain 4047) GN=SEQ_1906 PE=3 SV=1
MDTJ_SALA4 **Mass:** 12876 **Score:** 29 **Expect:** 6.5e+02 **Matches:** 2
Spermidine export protein mdtJ OS=Salmonella agona (strain SL483) GN=mdtJ PE=3 SV=1
MDTJ_SALCH **Mass:** 12906 **Score:** 29 **Expect:** 6.5e+02 **Matches:** 2
Spermidine export protein mdtJ OS=Salmonella choleraesuis GN=mdtJ PE=3 SV=1
MDTJ_SALDC **Mass:** 12906 **Score:** 29 **Expect:** 6.5e+02 **Matches:** 2
Spermidine export protein mdtJ OS=Salmonella dublin (strain CT_02021853) GN=mdtJ PE=3 SV=1
MDTJ_SALEP **Mass:** 12906 **Score:** 29 **Expect:** 6.5e+02 **Matches:** 2
Spermidine export protein mdtJ OS=Salmonella enteritidis PT4 (strain P125109) GN=mdtJ PE=3 SV=1
MDTJ_SALHS **Mass:** 12906 **Score:** 29 **Expect:** 6.5e+02 **Matches:** 2
Spermidine export protein mdtJ OS=Salmonella heidelberg (strain SL476) GN=mdtJ PE=3 SV=1
MDTJ_SALNS **Mass:** 12906 **Score:** 29 **Expect:** 6.5e+02 **Matches:** 2
Spermidine export protein mdtJ OS=Salmonella newport (strain SL254) GN=mdtJ PE=3 SV=1
MDTJ_SALPA **Mass:** 12804 **Score:** 29 **Expect:** 6.5e+02 **Matches:** 2
Spermidine export protein mdtJ OS=Salmonella paratyphi A GN=mdtJ PE=3 SV=1
MDTJ_SALPB **Mass:** 12906 **Score:** 29 **Expect:** 6.5e+02 **Matches:** 2
Spermidine export protein mdtJ OS=Salmonella paratyphi B (strain ATCC BAA-1250 / SPB7) GN=mdtJ PE=3 SV=1
MDTJ_SALPC **Mass:** 12906 **Score:** 29 **Expect:** 6.5e+02 **Matches:** 2
Spermidine export protein mdtJ OS=Salmonella paratyphi C (strain RKS4594) GN=mdtJ PE=3 SV=1
MDTJ_SALPK **Mass:** 12804 **Score:** 29 **Expect:** 6.5e+02 **Matches:** 2
Spermidine export protein mdtJ OS=Salmonella paratyphi A (strain AKU_12601) GN=mdtJ PE=3 SV=1

Search Parameters

Type of search : Peptide Mass Fingerprint
Enzyme : Trypsin
Mass values : Monoisotopic
Protein Mass : 100 kDa
Peptide Mass Tolerance : ± 20 ppm
Peptide Charge State : Mr
Max Missed Cleavages : 1
Number of queries : 21

Mascot: http://www.matrixscience.com/
